# Material-Dependent Effect of Heat Treatment on the Low-Velocity Impact Response of Additively Manufactured PA6 and PA6-CF Gyroid-Core Sandwich Structures

**DOI:** 10.3390/polym18212792

**Published:** 2026-11-08

**Authors:** Erkan Yılmaz

**Affiliations:** Department of Industrial Design Engineering, Engineering Faculty, Erciyes University, Kayseri 38039, Türkiye; erkan@erciyes.edu.tr

**Keywords:** additive manufacturing, gyroid, sandwich structure, low-velocity impact, energy absorption, heat treatment, PA6

## Abstract

Sandwich structures with gyroid cores produced via additive manufacturing show promise for lightweight impact-absorbing applications due to the stable plateau response provided by their continuous surface geometries; however, the effect of post-printing thermal history on the impact behavior of these structures has not been sufficiently investigated. In this study, three sandwich configurations were produced with 2 mm face sheets, a 10 mm core thickness, and a 10 mm gyroid cell size: all-PA6, all-PA6-CF, and a hybrid structure consisting of PA6-CF face sheets and a PA6 core. Non-heat-treated (Non-HT) and heat-treated (HT, 170 °C for 4 h) specimens were subjected to low-velocity impact at energy levels of 5, 10, 15, 20, and 25 J; peak force, absorbed energy, energy absorption efficiency (the ratio of absorbed energy to impact energy), and specific energy absorption (SEA) were compared. The effect of heat treatment depended on the material configuration and impact energy. DSC analyses showed that heat treatment increased the degree of crystallinity in PA6-CF from 6.4% to 11.4%, while in PA6, it increased only from 6.2% to 6.7%. In the PA6-CF configuration, heat treatment reduced the peak force by 28% and 34% at 15 and 20 J, respectively (8.41→6.04 kN; 8.20→5.42 kN), while increasing the absorbed energy from 17.5 J to 19.8 J at 20 J and giving the highest SEA of 0.243 J/g at 25 J. In the unreinforced PA6 configuration, heat treatment reduced the absorbed energy at 25 J from 15.7 J to 11.3 J and the energy absorption efficiency from 63% to 45%. The hybrid configuration exhibited the most stable response, with efficiencies of 92–101% in both cases within the 10–20 J range and limited peak forces in the 2.6–3.4 kN range. The findings indicate that post-printing heat treatment is an effective parameter for adjusting impact performance without changing the geometry or material, but that the direction of its effect depends on the presence of fiber reinforcement and the impact energy.

## 1. Introduction

In transportation, aerospace, and protective equipment applications, structural components are expected to offer both low mass and high energy absorption capacity. The geometric freedom of additive manufacturing has made it possible to produce cellular architectures that meet these expectations, and such structures have found a wide range of applications: energy-absorbing thin-walled and sandwich tubes with crash boxes [1,2,3,4,5,6], helmet liners and personal protective equipment [7,8], intermediate layers in shoe soles [9,10], bird-impact resistance in unmanned aerial vehicle structures [11], spinal implants [12], cement-based composite reinforcement [13], sound absorption [14], and vibration damping [15]. This widespread adoption has also brought with it a cautionary note that weight reduction does not always provide environmental benefits under all conditions and that the design algorithm has a dominant impact on production time [16].

In these structures, polyamides are among the most preferred engineering thermoplastics, and the predominant material is semi-crystalline PA12; there are numerous studies on its production via selective laser sintering [17,18,19,20,21], multi-jet fusion [22,23,24,25,26], and other powder-bed processes [27]. The key advantages of PA12 are high dimensional accuracy at low processing temperatures [17] and high impact strength combined with low density [28]. Other applications are limited: open-cell foams and filament development using PA6 [29,30], graded lattices using PA11 [31], and sandwich cores and reinforced auxetic structures using PA2200 [32,33]. In contrast, there is no systematic characterization of PA6-based lattices, TPMS, or sandwich structures; the closest reports involve structures produced using copolyamide 6/66-based filaments [34,35].

The response of cellular structures is primarily determined by the topology of the individual cells. Nasim and Galvanetto [25] compared eight topologies produced from the same base material and demonstrated that ranking them based on equal strut diameter or equal relative density resulted in different preference patterns. Prithvirajan et al. [18] determined that strut diameter is directly proportional to plateau stress and linear modulus, and inversely proportional to densification strain; Rodrigo et al. [31] and Sadeghzade et al. [36] demonstrated that the gradation of relative density and cell topology across layers directly controls the deformation pattern. Structures are classified as either bending-dominated or stretching-dominated; the former exhibits a long and stable plateau with high energy dissipation, while the latter exhibits high stiffness but softening after yielding [25,37]. Within this framework, auxetic and chiral derivatives [23,24], fractal architectures [27], stochastic Voronoi networks [29], and lattice column buckling [22] have been investigated. However, as Lazar et al. [35] emphasize, a common weakness of strut-based lattices is load fluctuations caused by stress concentrations at the nodes and sequential layer-wise collapse.

Triply periodic minimal surfaces (TPMSs) directly address this weakness: a continuous surface curvature improves the load distribution, suppresses stress peaks, and facilitates the transition to the plastic regime with a stable plateau stress [35]; Higuera et al. [38] have also noted that continuity eliminates the need for support. A distinctive feature of the gyroid within this class is its near-isotropy: Lambiase et al. [39] demonstrated that the anisotropy indices of three topologies fabricated from the same PA6-CF material varied between 1.0 and 3.1 depending solely on geometry; while the rectilinear pattern exhibited a 68% loss of properties under transverse loading, the gyroid remained within the 1.0–1.9 range, behaving nearly isotropically. Energy dissipation is also high; Higuera et al. [38] reported that PA12 sheet gyroid structures achieved a specific energy absorption (SEA) of 14.16 kJ/kg at a 25% volume fraction and retained their dissipation capability over the next two compression cycles, recovering 80% of their initial height after compression. Kepenekci et al. [34], on the other hand, demonstrated that the gyroid structure provided the smoothest plateau response and the highest energy absorption efficiencies in the 40–50% range compared to honeycomb and Voronoi structures. The same architecture has provided an improved stress distribution through continuous load paths as an infill pattern [15,40] and has been preferred in functional applications [9,10] and in various polymer systems [41].

The low strength of pure thermoplastics is a limiting factor in structural applications; short-fiber reinforcement is the most common solution. Kepenekci et al. [34] reported that carbon-fiber reinforcement increased the tensile strength of polyamide from 39.5 to 110.1 MPa (179%) and its modulus from 1.19 GPa to 9.85 GPa (725%), while Andrew et al. [28] reported that in PA12-CF15, the tensile strength increased from 45 to 120 MPa, and the modulus increased from 1400 to 7300 MPa. Similar improvements have been achieved in auxetic structures [42], isogrid shells [43], continuous glass-fiber-reinforced PA6 composites [44], and multi-material PA-CF/PA-GF structures [40]; at the cellular level, this has increased the yield stress approximately fourfold and the effective energy absorption by a factor of 2.2–5.8 [34]. However, these gains come at the expense of ductility: elongation at break has fallen below 2% in all raster orientations [34], and the elongation, which was 200% in PA12, has dropped to 5% in PA12-CF15 [28]. The structural consequences of this include brittle failure at low strain due to strut fracture, as observed by Garrido et al. [45]; limited post-impact protection due to reduced shape recovery [34]; increased anisotropy [35,39]; the transformation of interfacial delamination into the dominant failure mode in thin-walled sections, as identified by Hussain et al. [6]; and embrittlement at high strain rates, as demonstrated by Bandinelli et al. [46]. Indeed, Hao et al. [17] found that while glass fiber enhances the tensile properties of the ring lattice, it reduces its compressive load-bearing capacity and energy dissipation. Stiffness and toughness remain necessary yet conflicting properties [28].

Two families of hybrid structures have been developed to address this contradiction. In topological hybridization, different unit cells are combined in layers; Andrew et al. [28] demonstrated that hybrid hexagonal/re-entrant CF/PA12 honeycomb structures outperformed their single-layer counterparts of equal weight by factors of 2.83 in modulus, 1.64 in buckling resistance, and 2.25 in energy absorption; Bastola et al. [33] demonstrated that in reinforced hybrid auxetic–hexagonal PA2200 structures, the modulus increased by 168.26% and volumetric energy dissipation by 119.09%. In the second family, voids are filled with a secondary filler: Garrido et al. [45] and Valle et al. [47] demonstrated that polyurea infiltration shifts the collapse mode from brittle fracture to stable progressive collapse; it has been shown that powder, metal slurry, or foam fillers increase stiffness and SEA [48,49]; Rugerinyange et al. [50] reported that thermoset coating and graphene nanofillers increased the compressive yield strength by 201% and SEA by 154%. Foam injection and lattice-filled tubes also produce a synergistic effect [2,3,36]. However, this approach results in an increase in mass and may reduce the SEA [45]. Sandwich architecture offers a natural layered structure capable of providing the same decoupling without additional mass; however, performance is critically dependent on the face sheet–core interface. Ghasemi et al. [51] identified the bonding region as the primary source of experimental–numerical discrepancy in configurations where components were manufactured separately and bonded; Um et al. [52] found that a rail-locked joint provided an 18.8% increase in specific strength and a 22.9% increase in energy dissipation compared to the bonded model, and Elhassan et al. [53] found that slotted interlocking interfaces in monolithic-printed sandwich structures increased energy dissipation by 51%, while Solyaev et al. [32] demonstrated that changing the connection type in the core varied impact resistance by a factor of 3.5–5. The multilayer belt cores by Bao et al. [54] and the cylindrical shells by Johnson and Paramasivam [43] also confirm this interface sensitivity.

The overwhelming majority of the literature deals with quasi-static compression; however, loading rate is an independent source of uncertainty. Khosravani et al. [20] demonstrated, using split Hopkinson pressure bar tests on PA12 BCC and FCC lattices, that compressive strength increases with loading rate and that all specimens failed in a brittle manner under dynamic loading. Strain-rate sensitivity also depends on topology; Lambiase et al. [39] determined that the strength of gyroid and honeycomb structures increased by 10–25% at high relative densities, while the rectilinear pattern remained insensitive. The effect is not always positive: Bandinelli et al. [46] found that in short-fiber-reinforced polyamide structures, recycled material reduced the SEA under dynamic loading by 25%, whereas virgin material increased it by 22%, and they clearly emphasized that good quasi-static performance does not guarantee good high-speed performance. Similar findings, where the response diverges, have also been observed in the topology-driven lattices of Fang et al. [37], the fractal structures of Viccica et al. [7], the Voronoi networks of Cronau and Engstler [21], and in multiphase metamaterials [49]. Nevertheless, experimental data on the low-velocity impact response of 3D-printed polymer sandwich panels is limited: Ghasemi et al. [51] tested SLS PA12 lattice core and E-glass/epoxy-faced panels only at 12.4, 16.5, and 17.9 J levels, demonstrating that at least 90% of the impact energy was dissipated, while Solyaev et al. [32] subjected PA2200 sandwich beams to pendulum impacts in the 5.4–20 J range. Helmet [8], bird strike [11], and low-velocity collision [4] scenarios, on the other hand, are primarily based on numerical modeling.

Another parameter that determines performance in semi-crystalline polyamides is the degree of crystallinity. Lambiase et al. [39] demonstrated that the degree of crystallinity governs the matrix yield strength and, consequently, determines the upper limit of the plateau stress that can be achieved at a given relative density; higher crystallinity proportionally shifts the plateau stress upward, while amorphous regions contribute to rate sensitivity by dissipating energy through viscoelastic deformation. This relationship has also been experimentally confirmed; Guo et al. [30] demonstrated that PA6 and PA6/talc structures with a degree of crystallinity of approximately 24% exhibited SEA values of 113.63 and 130.02 J/g, respectively, whereas a commercial filament that remained largely amorphous upon cooling and had a degree of crystallinity of 10% remained at 32.51 J/g. Similarly, He et al. [42] attributed the lower performance of open-air-printed samples compared to their injection-molded counterparts to low crystallinity; Chen et al. [12] reported that strength increases as the melting and recrystallization rates rise in powder bed fusion. The crystal structure also changes with deformation: Bastola et al. [33] reported that polyamide transitions from the γ phase to the α phase under compression strains exceeding 30%, and that this reduces the elastic modulus, thereby affecting the repeated loading behavior. In addition, Xiong et al. [4] determined that carbon fiber acts as a heterogeneous nucleating agent, raising the crystallization temperature, and that at high concentrations, it restricts chain mobility, thereby altering the degree of crystallinity in a nonlinear manner; Wu et al. [13] demonstrated that the reinforcement contribution of 3D-printed polymers decreases as ambient temperature increases. These findings suggest that post-printing heat treatment could serve as a direct lever for tuning the mechanical response in polyamide-based cellular structures. In contrast, in the context of cellular (lattice, TPMS, and sandwich) structures, post-printing heat treatment has been applied only to metallic structures in the literature and has not been investigated in polymeric cellular structures: Bian et al. [55] annealed AlSi10Mg BCC lattice specimens by heating them to 280 °C and gradually cooling them to increase ductility, but did not report such a treatment for the PLA and PA12 specimens in the same study; Andrew et al. [28], on the other hand, explicitly state that no post-printing treatment was applied to the polymer structures.

In non-cellular, solid (dense) specimens, however, the situation is different: while post-printing heat treatment is an effective approach for modifying the mechanical performance of polyamide-based parts produced by FFF, results in the literature indicate that the effect of annealing depends significantly on the material composition and the applied temperature–time conditions [56,57,58,59,60]. In particular, it has been reported that appropriate annealing conditions can increase tensile strength and stiffness in carbon fiber-reinforced PA6 systems, although these gains may be accompanied by a loss of ductility in some cases [59,61]. Changes in mechanical behavior following heat treatment have been attributed, depending on the study, to alterations in fiber orientation, crystal structure and crystallization behavior, porosity, and interlaminar bonding [58,61,62]. However, annealing does not always produce positive results; while high temperatures or prolonged thermal exposure can lead to interfacial weakening, embrittlement, or a decrease in mechanical properties in some polyamide composites, appropriate temperature–time combinations can enable mechanical gains to be achieved while maintaining dimensional stability [56,57,60]. The fact that mechanical gains after annealing differ significantly in different matrix systems—such as PA12-CF and PETG-CF—and that the effect in PA-CF systems can vary depending on the test temperature and printing orientation indicates that the thermal treatment response cannot be evaluated independently of the material and loading conditions [63,64]. Similarly, it has been reported that post-printing heat treatment increases tensile strength in a different fiber/filler-reinforced PA12 system [65]. In semi-crystalline polyamides, thermal history is critical for understanding this behavior; the crystallization process can directly affect interlayer bonding and bond strength by altering macromolecular mobility and crystal morphology [66,67]. Therefore, the effect of post-printing annealing on polyamide-based additive manufacturing parts must be addressed not only through changes in the degree of crystallinity but also by evaluating changes in material composition, fiber reinforcement, porosity, interfacial behavior, and crystal morphology collectively [56,61,62,66,67].

In summary, there are five complementary gaps in the literature: the lack of systematic data on PA6-based cellular and sandwich structures; the fact that gyroid architecture has been studied primarily as standalone blocks and under quasi-static compression; the fact that hybridization has been addressed only through topological combination or void filling, and material-level decoupling of the face sheets and the core has not been attempted; the limitation of low-velocity impact data to a limited number of energy levels; and the fact that the effect of post-printing heat treatment on polymeric cellular structures has not yet been investigated. Aiming to address these gaps, this study investigates the low-velocity impact response and energy absorption behavior of gyroid-core sandwich structures under the combined influence of material configuration and post-printing heat treatment. Three configurations were fabricated, all with a 2 mm face-sheet thickness, a 10 mm core thickness, and a 10 mm gyroid cell size: all-PA6, all-PA6-CF, and a hybrid structure consisting of PA6-CF face sheets and a PA6 core. The hybrid configuration aims to prevent the embrittlement caused by the reinforcement from propagating to the core by utilizing the stiffness and strength gains provided by the fiber reinforcement solely in the face sheets, without additional mass. Each configuration was subjected to a low-velocity impact at energy levels of 5, 10, 15, 20, and 25 J; the same series of tests was then repeated for all configurations after heat treatment was applied. Thus, the material configuration, impact energy, and heat treatment condition are addressed in a full factorial design; the effect of heat treatment on peak force, contact time, absorbed energy, damage mode, and energy absorption efficiency can be isolated independently of the configuration.

The novelty of this study with respect to previous work can therefore be summarized as follows: First, previous studies on post-printing heat treatment of polyamides manufactured using material extrusion (MEX) have focused on solid (dense) specimens [56,57,58,59,60,61,62,63,64,65], whereas in cellular structures, heat treatment has been applied only to metallic lattices [55]; to the best of the author’s knowledge, this is the first study to investigate the effect of post-printing heat treatment on the low-velocity impact response of polymeric gyroid-core sandwich structures. Second, previous low-velocity impact studies on 3D-printed polymer sandwich structures [32,51] considered non-heat-treated specimens at a limited number of energy levels, whereas in this study, five impact energies (5–25 J) are evaluated in both the non-heat-treated and heat-treated conditions, allowing the effect of heat treatment to be tracked over a wide range of impact energies. Third, unlike topological hybridization [28,33] or void filling [45,47,48,49,50], the hybrid configuration proposed here decouples the face sheets and the core at the material level (PA6-CF face sheets and a PA6 core) without additional mass. Finally, by comparing unreinforced, fiber-reinforced, and hybrid configurations under identical heat treatment conditions, this study demonstrates that the effect of heat treatment on impact performance is material-dependent rather than universally beneficial, which is of practical relevance for selecting post-processing conditions for polyamide-based cellular structures.

## 2. Materials and Methods

### 2.1. Materials

In this study, two filaments were used to produce the sandwich panels by additive manufacturing: polyamide 6 (PA6) and PA6 containing 15% by weight carbon fiber (CF) (PA6-CF). Both materials were supplied in granular form and converted into filaments via single-screw extrusion. PA-based thermoplastics were selected because their semi-crystalline structure provides high toughness, good impact resistance, and a large plastic deformation capacity [39]. In PA6-CF, the short carbon fibers dispersed in the matrix increase the elastic modulus and strength. The technical properties of the PA6 and PA6-CF filaments are listed in Table 1.

### 2.2. Filament Fabrication

**Figure 1:**
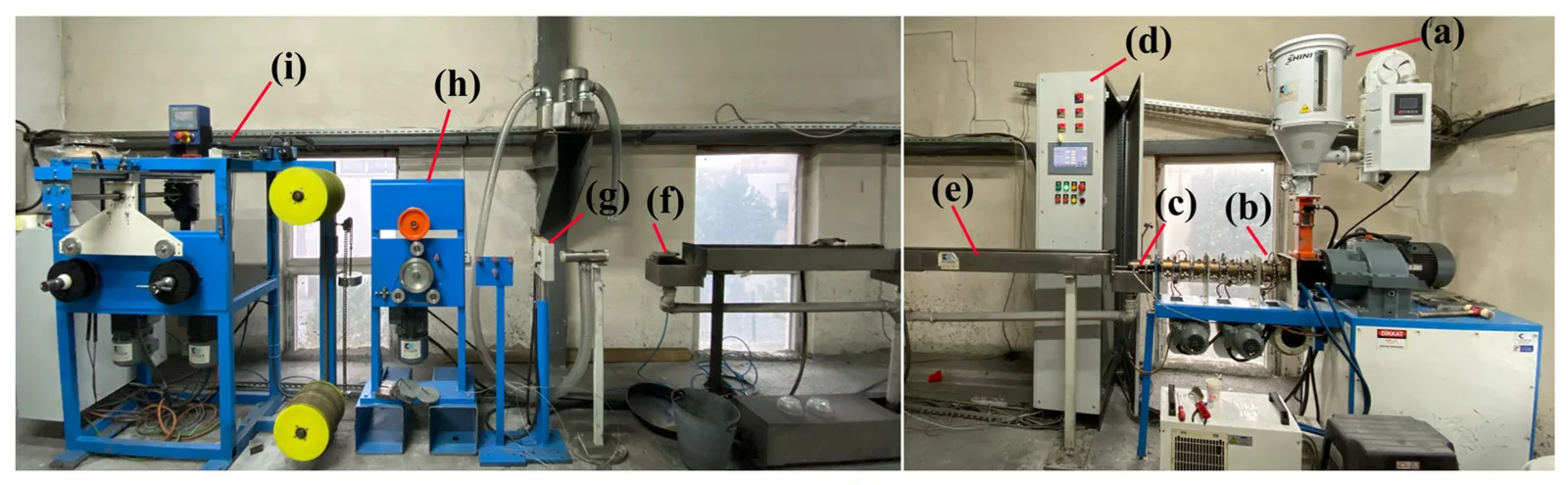
Polymer Filament Production Line Using a Single-Screw Extruder: (a) Raw Material Feeding and Air Drying; (b) Single-Screw Extruder; (c) Die; (d) Control Panel; (e) Cooling Bath; (f) Air Dryer; (g) Laser Diameter Gauge; (h) Filament Puller; (i) Filament Winder.

PA6 and PA6-CF filaments were produced on a single-screw extruder line, as shown in Figure 1. Because polyamides are strongly hygroscopic, the pellets were dried before extrusion. The PA6 pellets were dried at 90 °C for 12 h. PA6-CF pellets were dried in two stages: 90 °C for 12 h followed by 120 °C for 2 h. After drying, the pellets were transferred to the extruder feed zone and melted according to the regional temperature profile given in Table 2. The melt was conveyed by the screw and extruded through the die to form the filament. The filament emerging from the die was cooled by passing it sequentially through water baths at 65 °C and 25 °C. The filament diameter was monitored continuously by a closed-loop measurement unit and maintained at 1.75 mm.

Because carbon-fiber reinforcement increases the melt viscosity, the screw speed and feed rate were set higher than for the unreinforced material.

### 2.3. Additive Manufacturing of the Test Specimens

In this experimental study, the mechanical behavior of sandwich structures produced using the material extrusion (MEX) additive manufacturing process, specifically fused filament fabrication (FFF), was investigated under low-velocity impact loading. Each specimen consists of three layers: a bottom face sheet, a gyroid core, and a top face sheet. The face sheets were printed with 100% infill to distribute the impact load over the surface and to fully cover the core, while a gyroid with a 10 mm cell size was used for the core. This arrangement combines stiff load-bearing face sheets with a lightweight core of high energy absorption capacity. The sandwich panel was fabricated with external dimensions of 100 × 100 × 14 mm; each face sheet is 2 mm thick and the gyroid core is 10 mm thick.

The gyroid is a triply periodic minimal surface (TPMS) that repeats periodically in three-dimensional space. Unlike strut-based lattices, TPMS structures have a continuous, smooth surface without sharp junctions. The load is therefore distributed more uniformly and stress concentrations are reduced [35]. Changes in the unit cell size and relative density of the gyroid core alter its energy absorption capacity [38]. The gyroid core was not generated with the infill option of the slicing software; instead, it was modeled parametrically with a 10 mm cell size to obtain a relative density of 50% within a 100 × 100 × 10 mm block.

The gyroid surface defined by Schoen [69] can be approximated by an implicit level-set function [70]:

*φ*(*x*,*y*,*z*) = sin(*ω_x_x*)cos(*ω_y_y*) + sin(*ω_y_y*)cos(*ω_z_z*) + sin(*ω_z_z*)cos(*ω_x_x*) = *c* (1)

Here, *ω_i_* is the angular frequency that determines the periodicity along each axis and is related to the unit cell size *L_i_* as follows:

(2) $$\omega_{i}=\frac{2\pi }{L_{i}},\;i=x,y,z$$

In this study, the unit cell size was taken to be equal along all three axes (*L_x_* = *L_y_* = *L_z_* = 10 mm); therefore, *ω* = 2π/10 mm^−1^. For *c* = 0, Equation (1) yields the gyroid minimal surface, which has (approximately) zero mean curvature. To obtain a core of finite thickness, the region between two level sets offset symmetrically about the minimal surface was defined as solid, yielding a sheet-type gyroid:

*Ω*_solid_ = {(*x*,*y*,*z*)∈R^3^:−*c*≤*φ*(*x*,*y*,*z*)≤+*c*} (3)

The offset parameter *c* directly controls the relative density of the core. Relative density is defined as the ratio of the solid volume to the total block volume:

(4) $$\bar{\rho }=\frac{\rho *}{\rho_{s}}=\frac{V_{\mathrm{solid}}}{V_{\mathrm{total}}}$$

Here, ρ* is the apparent density of the core, and *ρ*_s_ is the bulk density of the filament material. For the core block with dimensions of 100 × 100 × 10 mm, the *c* parameter was iteratively adjusted so that the relative density $\bar{\rho }$, calculated using Equation (4), reached a value of 0.50.

**Figure 2:**
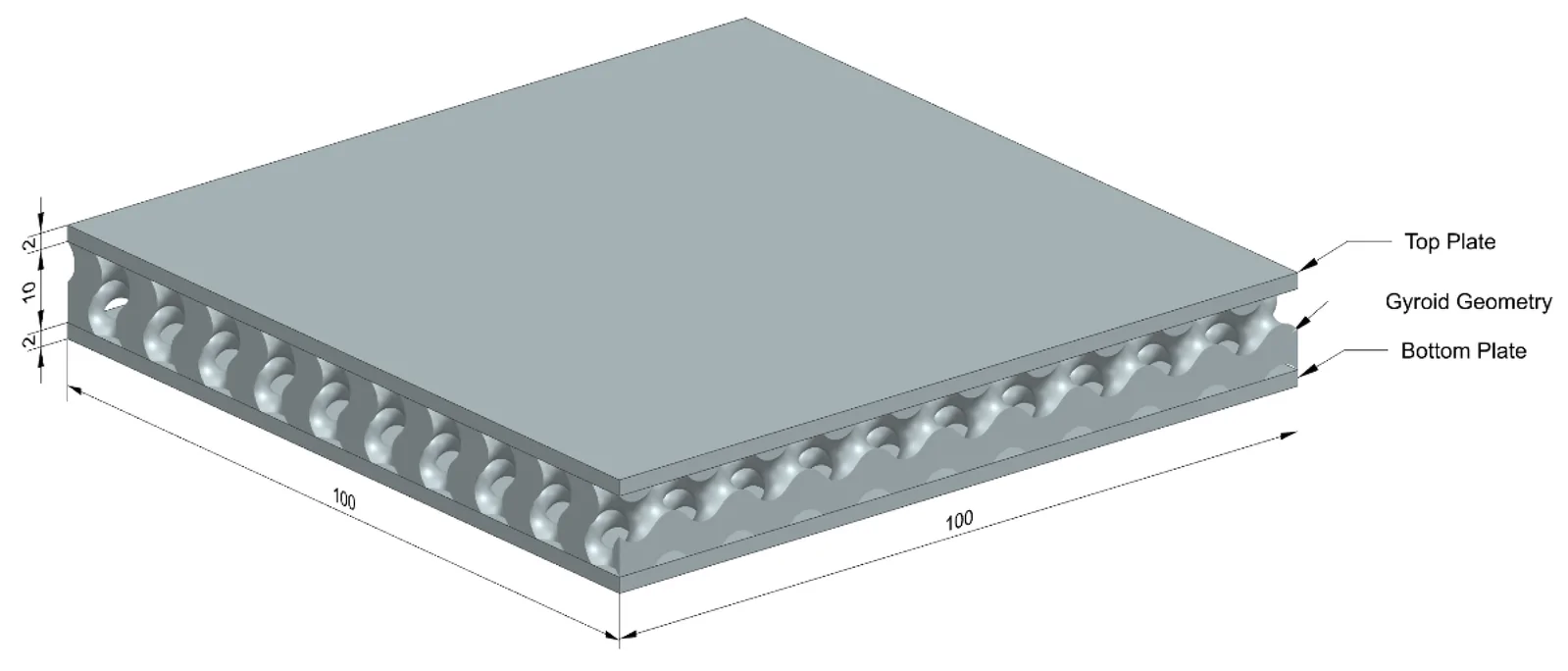
Schematic representation of the geometric configurations and specimen dimensions of the sandwich panels.

**Figure 3:**
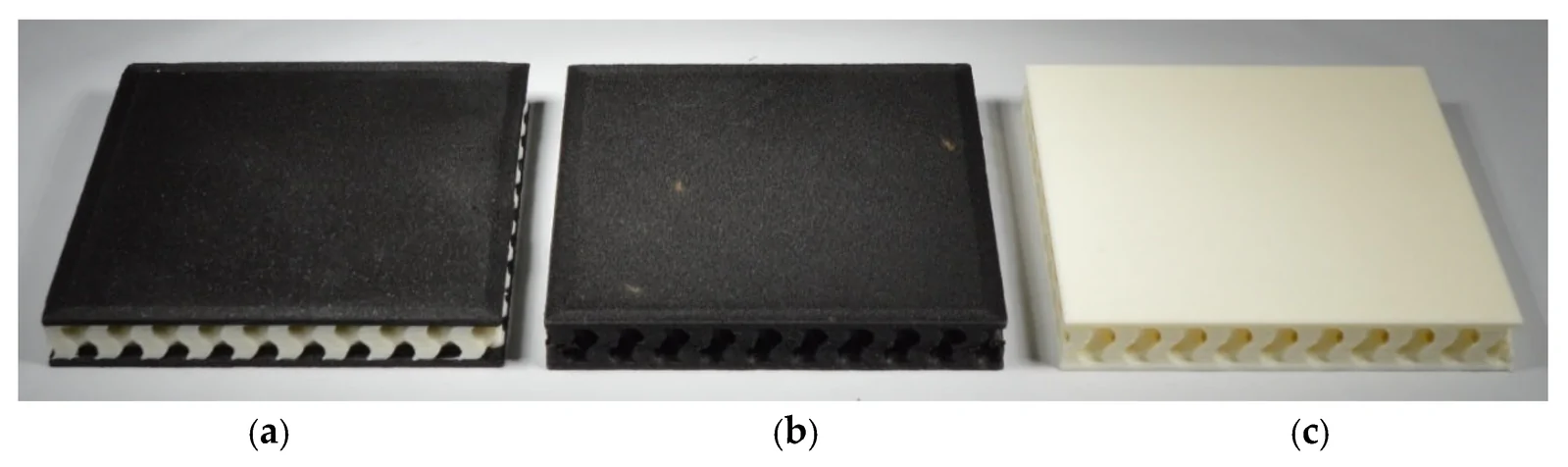
Test specimens of the sandwich panels: (**a**) hybrid panel; (**b**) PA6-CF panel; (**c**) PA6 panel.

Three different material models were designed to investigate the effects of material distribution on the low-velocity impact behavior (Table 3, Figure 2 and Figure 3). In the first model, all layers were fabricated from PA6 filaments. In the second model, all layers were fabricated from PA6-CF filaments. In the third model, a hybrid structure was created in which the face sheets were printed from PA6-CF and the core from PA6. This configuration was intended to provide stiffness, strength, and uniform load distribution in the face sheets, while the more compliant PA6 core absorbs energy through progressive deformation.

In addition to the mechanical properties of the polymer used in additive manufacturing, parameters such as print direction, layer thickness, nozzle diameter, and print speed also affect the mechanical behavior of the produced part. Although PA filaments offer high mechanical strength [71], they are more difficult to print than common filaments such as PLA and ABS and require higher bed (80–100 °C) and nozzle (240–290 °C) temperatures (Table 1). Owing to their hygroscopic nature, PA filaments must be dried before printing and kept dry throughout the process. Filaments that have not been sufficiently dried or that have absorbed moisture during printing cause vapor bubbles during extrusion, poor interlayer adhesion, and interrupted melt flow [56,72]. Print speed, part cooling, and chamber ventilation also affect print quality.

The printing parameters of PA6 and PA6-CF filaments differ due to their melting temperatures and flow behavior (Table 4). At high printing temperatures and long print times, the extruder assembly and the chamber heat up, and the filament may soften before reaching the hot end, resulting in filament jamming. For this reason, the part-cooling and chamber fan speeds were set separately for each material (Table 4).

The specimen models were sliced in Bambu Studio at 100% infill and printed on a Bambu Lab P2S printer. The printer features a closed print chamber, a hardened steel nozzle, and an all-metal hot end, making it suitable for printing filaments such as PA6-CF that are abrasive and require high temperatures. The bottom face sheet, gyroid core, and top face sheet of each specimen were printed sequentially in a single run. For the hybrid specimens, the filament was switched between layers without pausing the print. Since both filaments share the same PA6 matrix, the PA6 and PA6-CF layers were fused directly at the interface during printing, without any adhesive. No debonding or separation at the PA6/PA6-CF interfaces was observed in the damaged specimens after impact testing.

**Figure 4:**
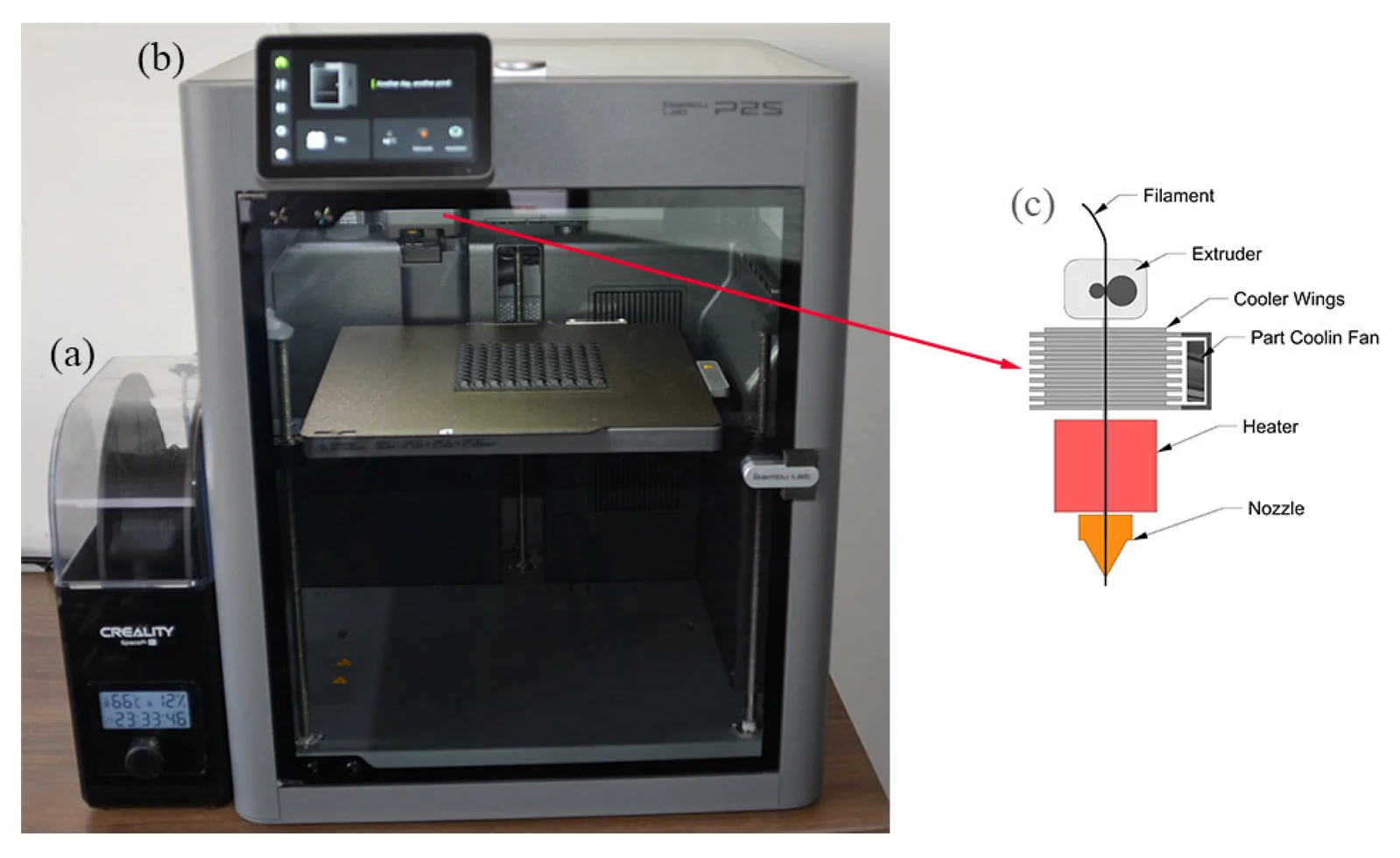
3D printing setup used for the fabrication of the test specimens: (**a**) filament dryer; (**b**) 3D printer; (**c**) schematic illustration of the extruder head.

Due to the hygroscopic behavior of PA-based filaments, both filaments were pre-dried at 65 °C for 8 h in a filament dryer (Figure 4a), and drying was continued throughout printing. The filaments were fed from the dryer to the printer through a PTFE tube to limit exposure to ambient humidity. A 0.4 mm nozzle and a layer thickness of 0.2 mm were used for the prints. Since the melting temperature of the PA6 filament is lower than that of the PA6-CF filament, the part-cooling fan was set to automatic and the chamber fan to 60%. For PA6-CF, both fans were switched off.

To improve bed adhesion and prevent the parts from lifting, an adhesive developed for 3D printing was applied to the build plate before printing; this adhesive activates at temperatures above 50 °C. Before starting the printing process, the chamber temperature was brought to 60 °C and the bed temperature to 100 °C, and both were maintained throughout printing. After printing was completed, the chamber was cooled gradually to 25–30 °C to prevent warping due to thermal stresses.

### 2.4. Heat Treatment (Annealing)

**Figure 5:**
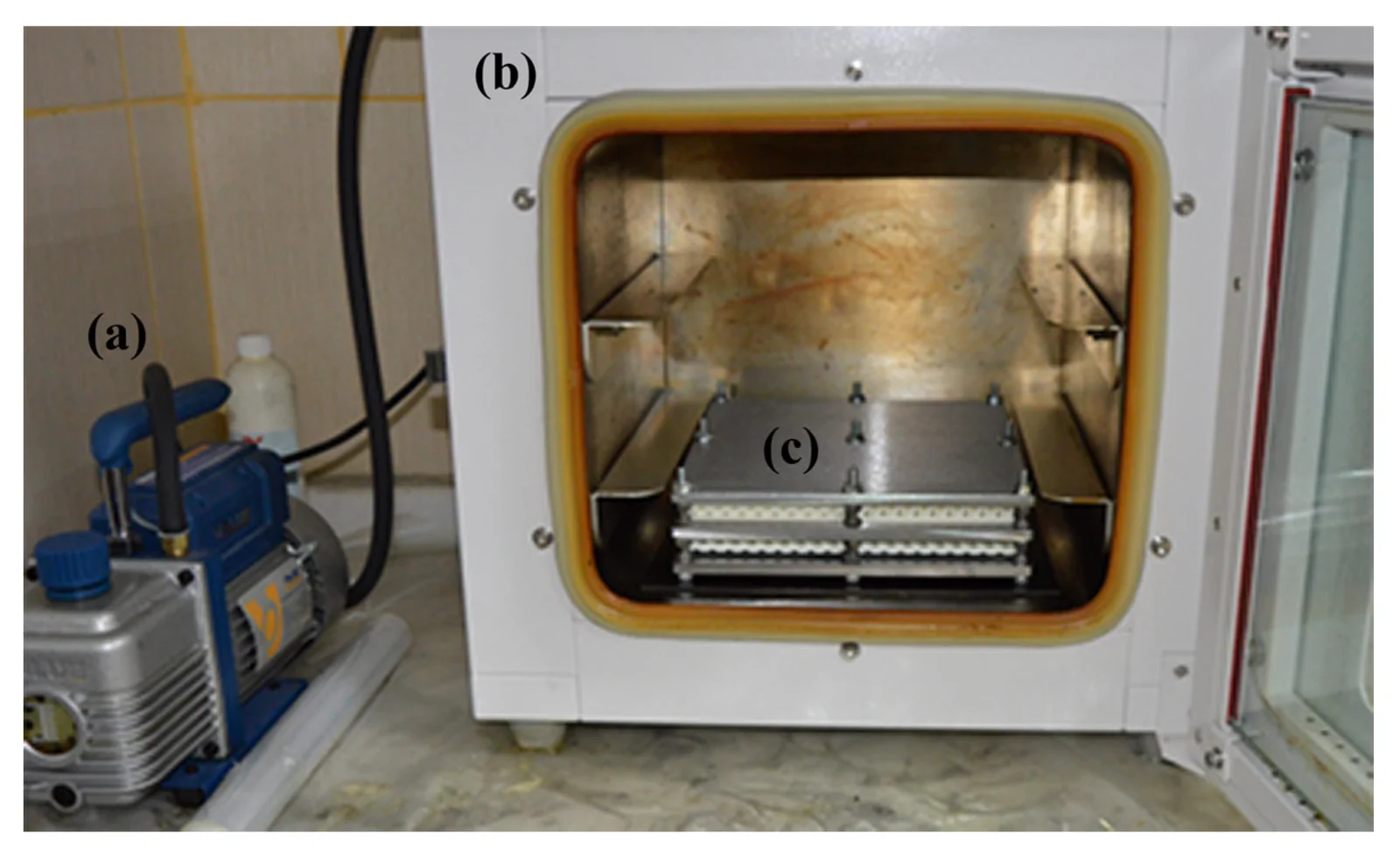
Drying and annealing setup: (**a**) vacuum pump, (**b**) heat treatment furnace, (**c**) molds and test specimens.

Annealing has been reported to increase the tensile strength of MEX -printed PA-based parts [73]. Similarly, Rahmatabadi et al. reported that annealing short carbon fiber-reinforced PETG parts in a metallic mold under vacuum improved their shape memory performance and reduced the layered structure and micro-voids formed during printing [74]. Because absorbed moisture strongly affects the mechanical properties of polyamides, the specimens were dried before heat treatment. Drying was carried out under vacuum at 70 °C for 8 h [56]. The specimens were then heat-treated (HT) at 170 °C for 4 h, i.e., between the glass transition and melting temperatures, without breaking the vacuum [59]. After annealing, the specimens were cooled inside the oven under vacuum until the temperature dropped to 28 °C, and were then removed from the oven. During drying and annealing, the specimens were held in molds (Figure 5c) to prevent warping during heating and cooling. The Non-HT specimens did not undergo this vacuum-drying step; however, they were printed from filaments that had been dried and kept dry throughout printing. The Non-HT condition was intentionally selected to represent the as-printed state in which MEX parts are commonly used in practice, whereas the HT condition represents the complete post-processing route (vacuum drying followed by annealing). Therefore, the differences between the two conditions reflect the combined effect of this post-processing route rather than the effect of annealing alone. After printing or heat treatment, all specimens were stored under laboratory conditions and tested within 24 h.

**Figure 6:**
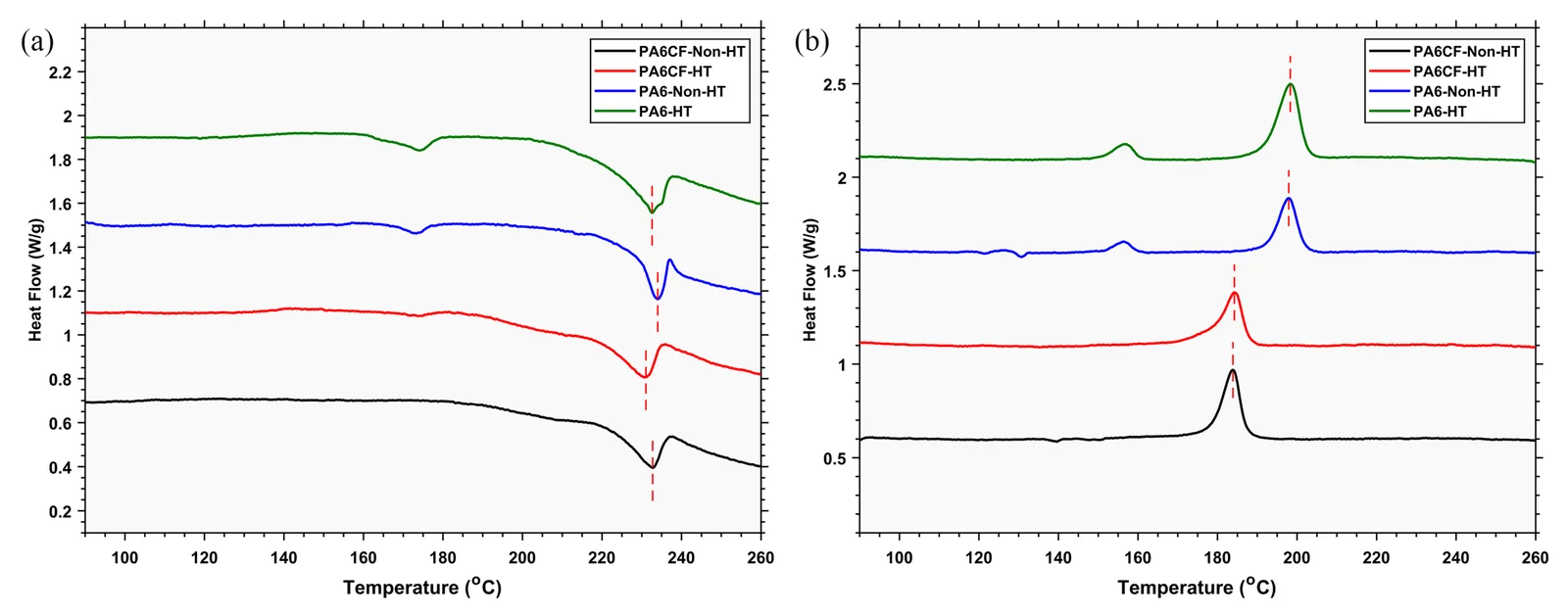
DSC curves of PA6-Non-HT, PA6-HT, PA6-CF-Non-HT and PA6-CF-HT specimens: (**a**) heating; (**b**) cooling. The dashed lines indicate the peak positions and are included to allow the peaks of the different specimens to be clearly distinguished.

To investigate the effects of carbon-fiber reinforcement and the heat treatment process on the material, DSC analysis was performed on non-heat-treated PA6 (PA6-Non-HT), heat-treated PA6 (PA6-HT), non-heat-treated PA6-CF (PA6-CF-Non-HT), and heat-treated PA6-CF (PA6-CF-HT) samples. The measurements were carried out using a simultaneous thermal analyzer (NEXTA STA 300, Hitachi, Japan) at the Central Research Laboratory of İskenderun Technical University (İSTE-BTM) from room temperature to 280 °C under a nitrogen atmosphere at a heating and cooling rate of 5 °C/min, using specimens with a mass of approximately 8 mg for all conditions. One specimen was measured for each condition, and the measurement was repeated when an inconsistent result was obtained. The heating and cooling curves are shown in Figure 6. The melting temperature of all specimens remained within a narrow range (Tm = 231–237 °C), indicating that the carbon-fiber reinforcement and annealing do not significantly alter the melting temperature [75,76]. Because the first heating scan melts the specimen and erases its processing and thermal history, the crystallization behavior in the subsequent cooling scan reflects the material composition rather than the heat treatment. Differences in the crystallization temperature (Tc) indicate that the carbon fiber addition affects the crystallization kinetics. Tc was lower in carbon fiber-reinforced specimens (PA6-CF, Tc ≈ 184 °C) than in the unreinforced specimens (PA6, Tc ≈ 198 °C), suggesting that the carbon fibers delay the onset of crystallization during cooling from the melt [77]. It should be noted that this Tc reflects non-isothermal crystallization from the melt at a constant cooling rate, whereas Xc in Table 5 was obtained from the first heating scan and therefore reflects the Non-HT and HT states. During printing, the deposited layers cool rapidly, and the retarded crystallization of the PA6-CF matrix leaves a larger fraction of crystallizable amorphous material in the Non-HT specimens. During annealing at 170 °C, which lies between the glass transition and melting temperatures, sufficient time and chain mobility are provided for this fraction to crystallize, and the fiber surfaces may additionally act as nucleation sites under these isothermal conditions. Therefore, the two observations refer to different crystallization conditions and are not contradictory.

The degree of crystallinity (Xc) was calculated from the melting endotherm of the first heating scan using Equation (5). Since no cold-crystallization exotherm was observed in the first heating scans, the measured melting enthalpy was used directly as ΔHm. Annealing increased Xc in both PA6 and PA6-CF, and the increase was larger in PA6-CF (Table 5).

(5) $$X_{c}=\frac{{∆H}_{m}}{(1-W_{f}){∆H}_{100}}\times 100$$

where ΔHm is the measured enthalpy of fusion, Wf is the mass fraction of the carbon-fiber reinforcement (0.15 for PA6-CF and 0 for neat PA6), and ∆*H*_100_ is the enthalpy of fusion of 100% crystalline PA6 (230 J g^−1^) [75].

### 2.5. Low-Velocity Impact Testing

**Figure 7:**
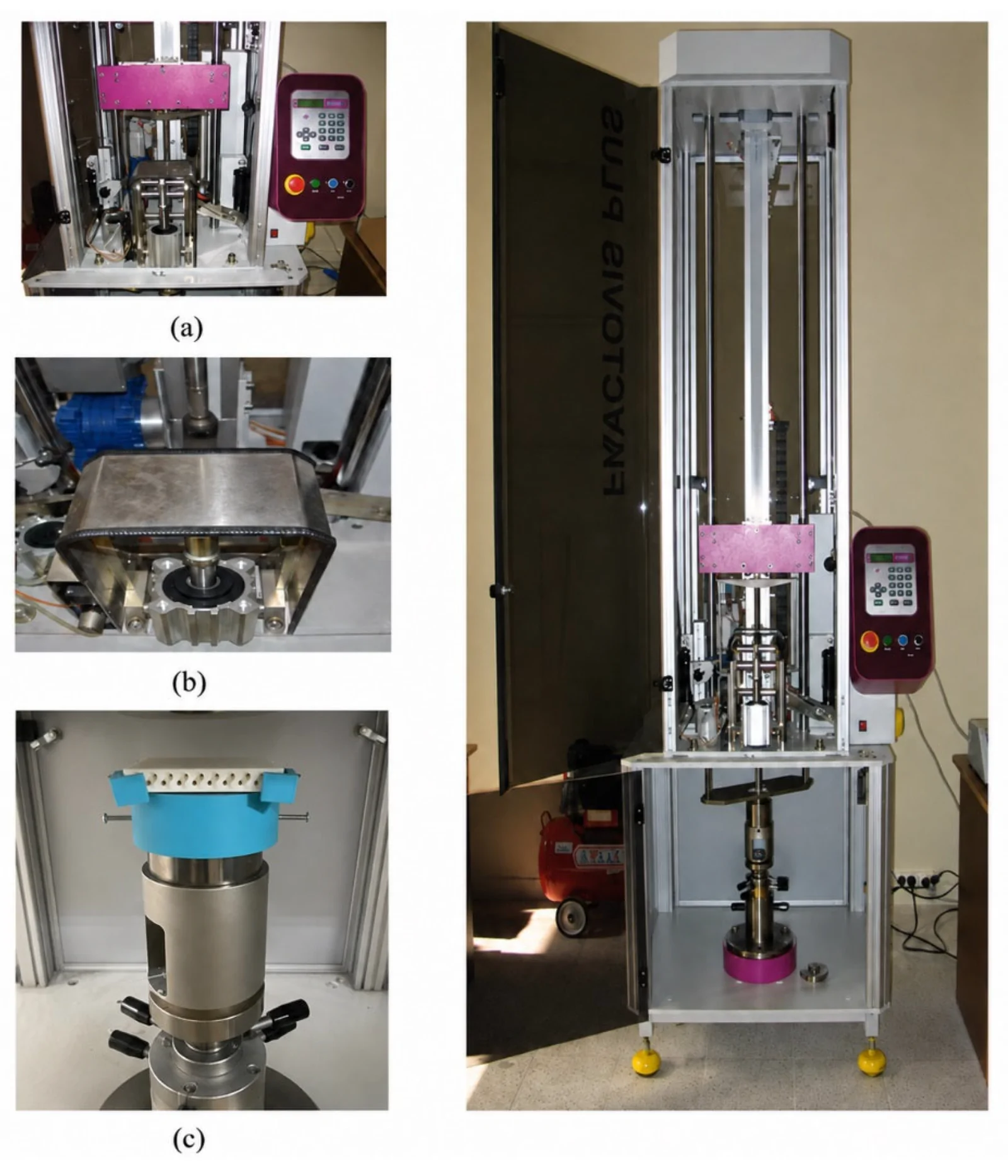
Experimental setup of the CEAST Fractovis drop-weight impact testing system: (**a**) electronic control unit, (**b**) impactor assembly and braking mechanism, and (**c**) specimen clamping fixture.

Low-velocity impact (LVI) tests were carried out on the fabricated sandwich specimens with gyroid cellular cores using a CEAST Fractovis drop-weight impact testing machine located in the Mechanical Engineering Laboratories of Erciyes University in accordance with ISO 6603-2 [78]. The testing system is equipped with an anti-rebound mechanism that prevents the impactor from striking the specimen multiple times after the first contact, thereby ensuring a single-impact event. During each test, the impact response was continuously recorded from the initial contact until the end of the impact event. The apparatus can operate within an impact velocity range of 0.8–25 m s^−1^. A hemispherical impactor tip with a diameter of 20 mm was used, and the specimens were clamped in a pneumatic fixture during testing. The pneumatic fixture had a circular support opening with an inner diameter of 40 mm and applied a clamping force of 2400 N to the specimen from above. Each specimen was placed in the centering fixture (Figure 7c) so that the impact point coincided with the center of the specimen; before each test, the impactor was lowered to its zero position, and the height of the lower support mechanism was adjusted until the impactor tip just contacted the specimen surface. The contact force was measured with an instrumented impactor equipped with a 60 kN force sensor, and the impact velocity was measured by a velocity sensor at the moment of contact (zero position). The force–time data were recorded using the CEAST DAS data acquisition system, and no filtering was applied to the signals. Since the system records only the force–time history and the initial impact velocity, the impactor velocity and displacement were obtained by successive integration of the equation of motion in accordance with ISO 6603-2, i.e., v(t) = v_0_ + gt − (1/m)∫F dt and δ(t) = ∫v dt, where v_0_ is the measured initial impact velocity, g is the gravitational acceleration, and m is the impactor mass. A constant impactor mass of 5.295 kg was used, and the specimens were tested at five different impact energy levels: 5, 10, 15, 20, and 25 J. For each material configuration, heat treatment condition and impact energy level, at least two specimens were tested; when a noticeable discrepancy was observed between the two results, a third specimen was tested. The curves and values presented in Section 3 correspond to the representative specimens whose damage images are shown. The replicate specimens exhibited similar force–time responses and damage modes, and the trends discussed in Section 3 were consistent across replicates. Therefore, the percentage changes reported in this study refer to the representative specimens and should be regarded as indicative of the observed trends rather than as statistically established values. The corresponding impact velocities, calculated from the energy–velocity relation E = ½mv^2^, are summarized in Table 6.

As shown in Figure 7, the testing system consists of a rigid base frame supporting the specimen fixture, vertical steel guide rails, a drop-weight assembly, a connection mechanism between the guide rails and the impactor, a specimen clamping fixture, and an electronic control unit. A pulley mechanism is employed to raise the impact mass to the required height, while a mechanical release hook securely holds the impactor before testing. Once the release mechanism is actuated, the impact mass is released and falls freely under gravity along the vertical steel guide rails until it strikes the specimen mounted on the fixture at the prescribed impact energy. This setup was used to evaluate the low-velocity impact behavior of the heat-treated (HT) and non-heat-treated (Non-HT) PA6, PA6-CF, and hybrid gyroid-core sandwich structures. The force and displacement data recorded during each test were converted to kN and mm, respectively, and the absorbed energy (E_abs_) was calculated as the area under the force–displacement curve using trapezoidal numerical integration (trapz function, MATLAB R2023a, The MathWorks Inc., Natick, MA, USA):

(6) $$E_{\mathrm{abs}}=\int Fd\delta \approx \sum_{k=1}^{n-1}\frac{F_{k}+F_{k+1}}{2}\left(\delta_{k+1}-\delta_{k}\right)$$

where F is the contact force (kN), δ is the impactor displacement (mm), k is the data-point index, and n is the number of recorded data points. Since 1 kN × 1 mm = 1 J, Equation (6) directly yields the absorbed energy in joules. The integration was performed over the complete loading–unloading history, from the initial contact until the impactor separated from the specimen. During rebound, the displacement decreases and the corresponding increments are negative; therefore, the elastic energy returned to the impactor is automatically subtracted, and E_abs_ represents only the energy dissipated by the specimen. The same cumulative integration was used to obtain the energy–time histories in Section 3, where the peak value corresponds to the maximum energy transferred to the specimen and the final plateau corresponds to E_abs_. The energy absorption efficiency (%EA), which represents the percentage of the nominal impact energy (E_nominal_) dissipated by the specimen, was calculated as follows:

(7) $$\%\mathrm{EA}=\left(\frac{E_{\mathrm{abs}}}{E_{\mathrm{nominal}}}\right)\times 100$$

Since E_nominal_ does not include the additional work done by gravity on the impactor during specimen deformation (mgδ ≈ 0.5 J for a displacement of 10 mm), the %EA values of a few hybrid specimens slightly exceeded 100% (up to approximately 101%). These values do not indicate that the specimen absorbed more energy than was delivered; the total energy transferred to the specimen is the sum of the kinetic energy at contact and the work done by gravity during deformation (Etotal = ½mv_0_^2^ + mgδmax), and the absorbed energy remained below this total in all tests. The specific energy absorption (SEA), which enables specimens of different masses to be compared on a per-unit-mass basis, was calculated as the ratio of the absorbed energy to the specimen mass:

(8) $$\mathrm{SEA}=\frac{E_{\mathrm{abs}}}{m}$$

where m is the specimen mass (g), so that SEA is expressed in J/g. The measured specimen masses were 95 g for PA6, 98 g for the hybrid configuration, and 103 g for PA6-CF; since annealing did not cause any measurable change in specimen mass, the same values were used for the HT and Non-HT conditions.

## 3. Results

In this section, the low-velocity impact responses of PA6, PA6-CF, and hybrid gyroid-core sandwich panels at 5, 10, 15, 20, and 25 J are compared in the non-heat-treated (Non-HT) and heat-treated (HT) conditions. Peak force and contact time were evaluated from the force–time and force–displacement curves, and absorbed energy and energy absorption efficiency from the energy–time curves obtained by cumulative integration of the force–displacement data (Equations (6) and (7)). Damage modes were identified from images of the front and back faces at each energy level, and the configurations were compared in terms of specific energy absorption (SEA). This sequence allows the individual and combined effects of material configuration, impact energy, and heat treatment to be distinguished.

**Figure 8:**
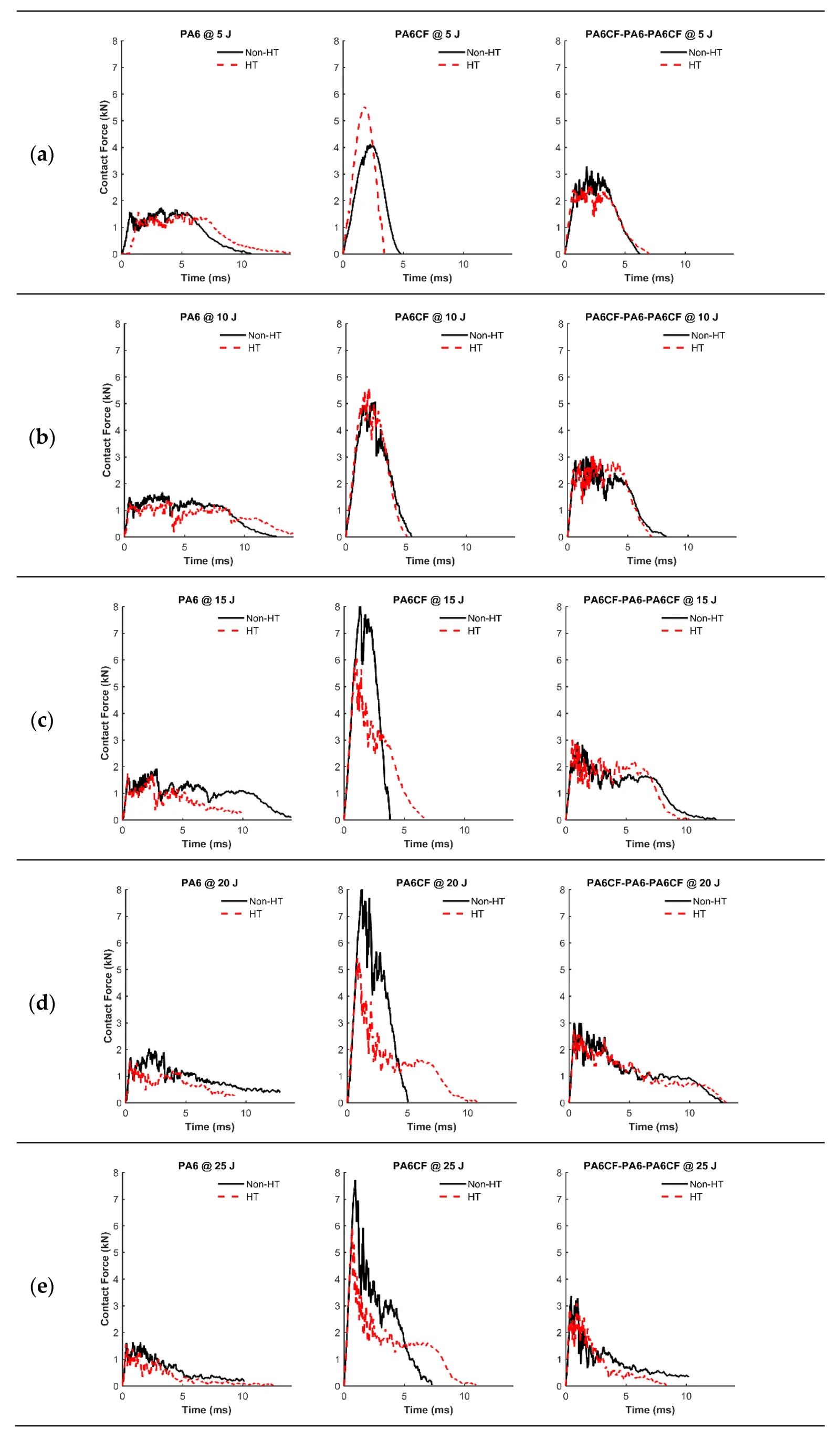
Contact force–time responses of the three sandwich configurations in non-heat-treated (Non-HT) and heat-treated (HT) conditions: (**a**) 5 J, (**b**) 10 J, (**c**) 15 J, (**d**) 20 J, (**e**) 25 J.

Figure 8 shows the contact force–time responses of PA6, PA6-CF, and hybrid sandwich structures at 5–25 J in the Non-HT and HT conditions. The PA6 specimens showed low peak forces (approximately 1–2 kN) and relatively long contact times at all energy levels; the broad, oscillating force–time profile indicates that the load was carried by progressive deformation rather than sudden fracture. The PA6-CF specimens showed the highest peak forces and the shortest contact times, particularly at 15–25 J. Sharp force peaks reaching approximately 8 kN in Non-HT PA6-CF specimens indicate high stiffness and a more brittle impact behavior. In PA6-CF, heat treatment slightly increased the peak force at low energies but markedly reduced it at 15–25 J and extended the contact duration, which is consistent with less abrupt damage development and more progressive deformation. The hybrid panels combined lower peak forces (approximately 2–3 kN) with long contact times, giving a less brittle response than PA6-CF and a more controlled response than PA6. Heat treatment had only a limited effect on the hybrid response.

**Figure 9:**
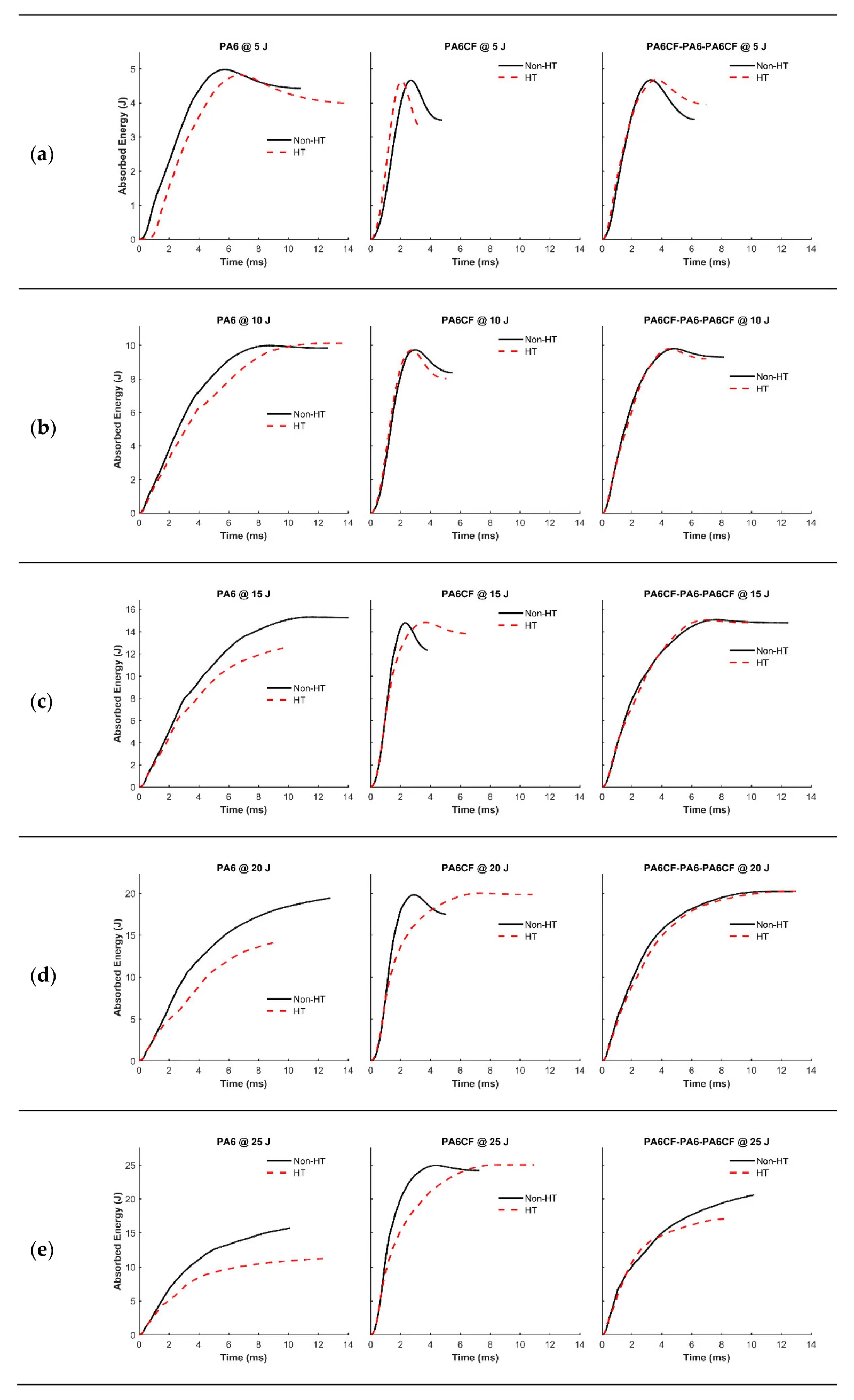
Absorbed energy–time histories: (**a**) 5 J, (**b**) 10 J, (**c**) 15 J, (**d**) 20 J, (**e**) 25 J. The plateau value denotes the total absorbed energy.

Figure 9 shows the absorbed energy–time histories of the PA6, PA6-CF, and hybrid sandwich structures at 5–25 J. In the PA6 specimens, the energy increased over a longer time interval, indicating ductile energy absorption accompanied by larger deformation. Heat treatment reduced the final absorbed energy of PA6, particularly at 15–25 J; at 25 J, the value decreased from approximately 15.7 J to 11.3 J. In the PA6-CF specimens, the energy rose much more rapidly, consistent with the higher stiffness and faster load transfer provided by the carbon fibers. The decrease after the maximum corresponds to the elastic energy returned to the impactor during rebound, and the final plateau represents the energy absorbed by the specimen. In PA6-CF, heat treatment increased the absorbed energy at 20 J from approximately 17.5 J to 19.8 J. The HT and Non-HT hybrid panels showed nearly identical energy histories at 5–20 J, whereas at 25 J, the absorbed energy decreased after heat treatment. Energy absorption in PA6 is slower and deformation-dominated, in PA6-CF it is faster and stiffness-dominated, and the hybrid lies between the two.

**Figure 10:**
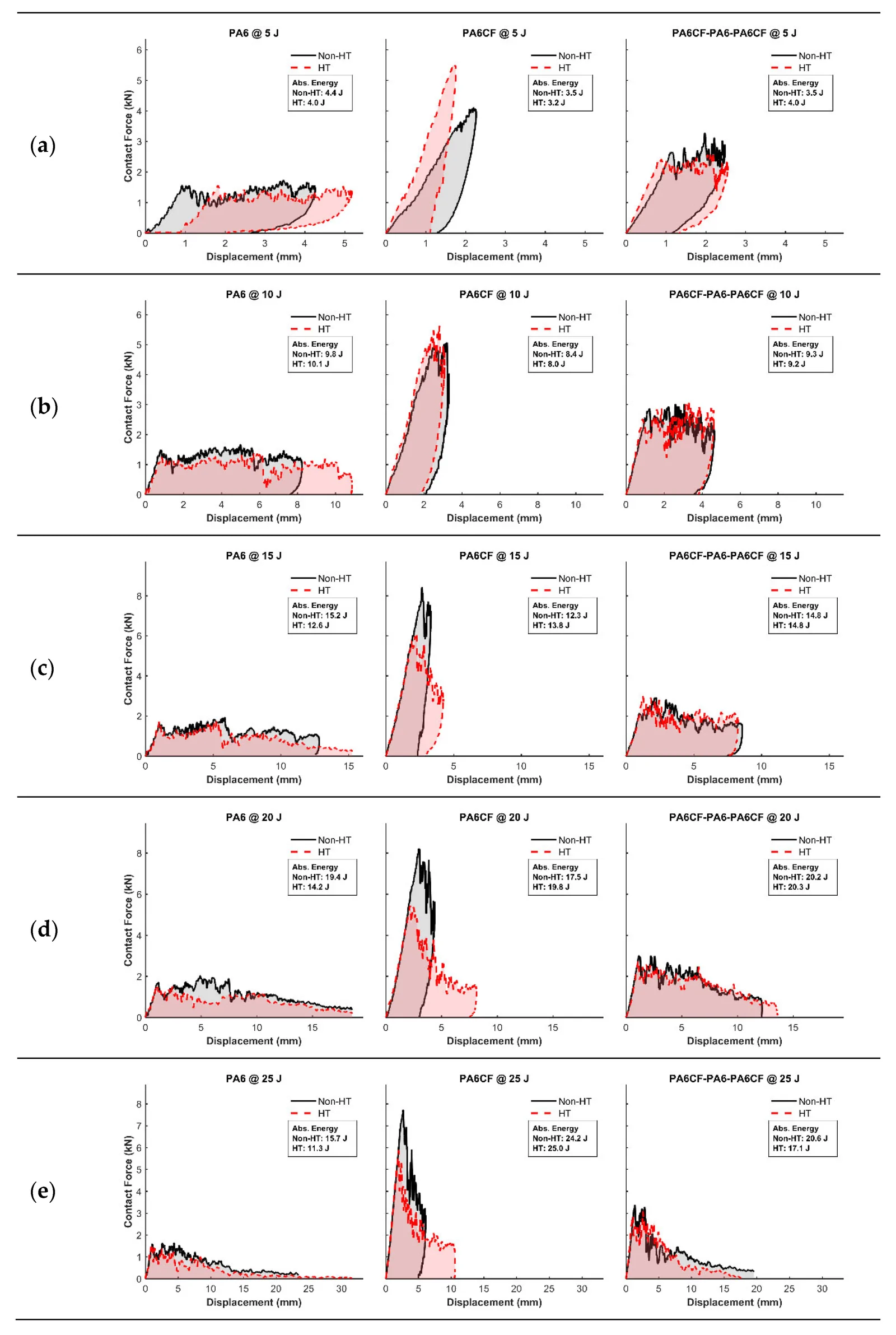
Contact force–displacement curves with the corresponding absorbed energy values: (**a**) 5 J, (**b**) 10 J, (**c**) 15 J, (**d**) 20 J, (**e**) 25 J.

Figure 10 shows the contact force–displacement behavior of PA6, PA6-CF, and hybrid sandwich structures at 5–25 J together with the corresponding absorbed energies. The PA6 specimens showed larger displacements at lower peak forces, indicating more widespread, ductile deformation. In the PA6-CF specimens, the force rose steeply over a smaller displacement and sharper peaks formed, particularly at 15–25 J; this behavior is consistent with high stiffness and a more brittle load-bearing character. Heat treatment reduced the peak force of PA6-CF and increased its maximum displacement, raising the absorbed energy from 17.5 J to 19.8 J at 20 J and from 24.2 J to 25.0 J at 25 J. This apparently contradictory behavior follows from Equation (6): the absorbed energy is determined by the area under the force–displacement curve rather than by the peak force alone. In the Non-HT PA6-CF panels, the force rose steeply to a high peak and then dropped abruptly, indicating that the stiff structure failed suddenly over a small displacement and returned a larger share of the impact energy to the impactor. After heat treatment, the higher crystallinity (from 6.4% to 11.4%, Table 5) and the relief of residual stresses introduced during printing may have contributed to a more progressive damage process, possibly involving the gradual development of matrix cracking, fiber–matrix debonding, and fiber pull-out rather than a single abrupt failure. Consequently, the load was carried at a lower but more sustained force level over a larger displacement and a longer contact time, which increased the area under the force–displacement curve and reduced the elastic energy returned to the impactor during rebound. In PA6, heat treatment had the opposite effect at high energies; the energy absorbed at 20 J decreased from 19.4 J to 14.2 J, and at 25 J, it decreased from 15.7 J to 11.3 J. At 15 and 20 J, the hybrid panels combined moderate forces with large displacements and absorbed approximately 20 J at 20 J in both conditions. The force–displacement curves therefore reflect a ductility-dominated response for PA6, a stiffness-dominated response for PA6-CF, and an intermediate response for the hybrid.

**Figure 11:**
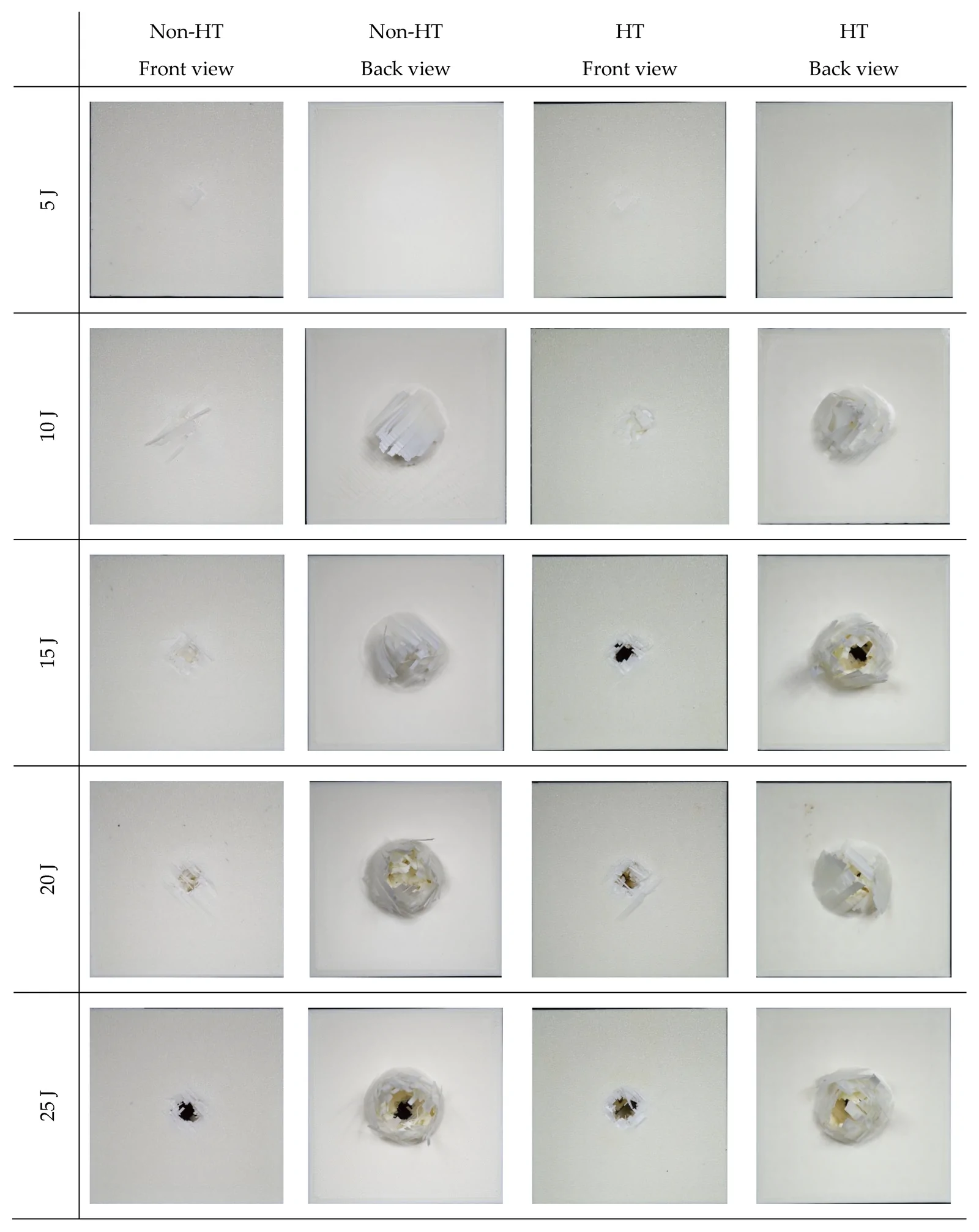
Impact damage morphology of the PA6 sandwich panels in the non-heat-treated (Non-HT) and heat-treated (HT) conditions; front and back views at impact energies of 5, 10, 15, 20, and 25 J.

Figure 11 shows the front- and back-face damage of the PA6 sandwich panels at 5–25 J in the Non-HT and HT conditions. No visible permanent damage was observed at 5 J, whereas from 10 J onward, more extensive plastic deformation and local openings appeared on the back face. At 15–20 J, local cracking developed on the front face, and bulging, tearing, and petalling developed on the back face. PA6 therefore absorbs the impact energy through plastic deformation that spreads beyond the impact point. In the HT specimens, front-face damage became more localized and pronounced at 15 J and above, indicating that deformation was concentrated in a smaller region. At 25 J, penetration occurred in both conditions, while the large deformed area on the back face reflects the high deformation capacity of PA6. The more localized fracture and lower absorbed energy of the HT specimens at high energies suggest that heat treatment reduces the ductile energy absorption capacity of PA6.

**Figure 12:**
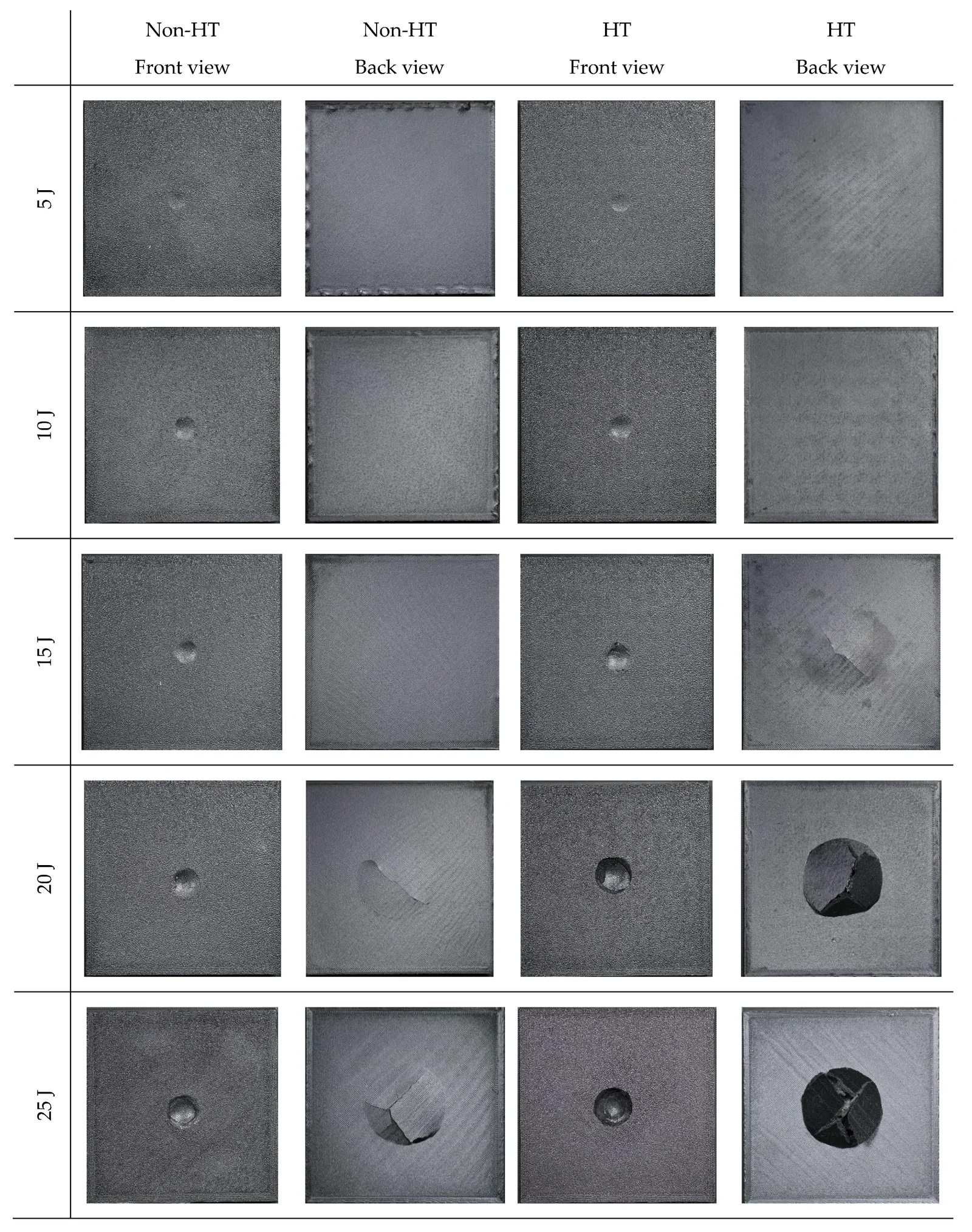
Impact damage morphology of the PA6-CF sandwich panels in the non-heat-treated (Non-HT) and heat-treated (HT) conditions; front and back views at impact energies of 5, 10, 15, 20, and 25 J.

Figure 12 shows the front- and back-face damage of the PA6-CF sandwich panels at 5–25 J in the Non-HT and HT conditions. At 5 and 10 J, damage remained confined to the impact zone, and no back-face fracture was observed. At 15 J, a distinct local deformation zone formed on the back face, particularly in the HT specimens, and at 20 and 25 J the damage changed to penetration with local fracture. At high energies, the back faces of the Non-HT specimens showed wider petal-like openings and layer deformation, whereas the HT specimens showed sharply defined circular openings and radial cracks. This morphology indicates that damage in the PA6-CF structure became more localized following heat treatment, with sharper fracture edges at high energy levels. The higher absorbed energy of the HT PA6-CF specimens at 20 and 25 J shows that this localized fracture does not reduce energy absorption; the additional energy is presumably dissipated during penetration through mechanisms such as fiber–matrix interfacial damage and crack propagation. This localization is supported by the damage morphology in Figure 12: in the HT specimens, the back-face damage was confined to a sharply defined circular opening with radial cracks around the impact point, whereas the Non-HT specimens showed wider petal-like openings and layer deformation extending further from the impact point. It is also consistent with the measured impact response. The lower peak force of the HT PA6-CF panels at 15–25 J suggests that a smaller part of the surrounding panel was engaged in carrying the load, while the larger maximum displacement and longer contact time (Figure 8 and Figure 10) show that the impactor was resisted over a longer penetration path. Thus, heat treatment concentrated the damage in the plane of the panel but extended it through the thickness, where energy was presumably dissipated by core crushing, crack propagation, and possibly fiber–matrix debonding along the penetration path; this resulted in a higher absorbed energy and less energy returned to the impactor during rebound. It should be noted that no microscopic fractographic analysis (e.g., SEM) was performed in this study; therefore, the fiber–matrix interfacial damage and crack propagation mechanisms discussed above were inferred from the macroscopic damage morphology and the force–displacement response and should be regarded as plausible explanations rather than confirmed observations.

**Figure 13:**
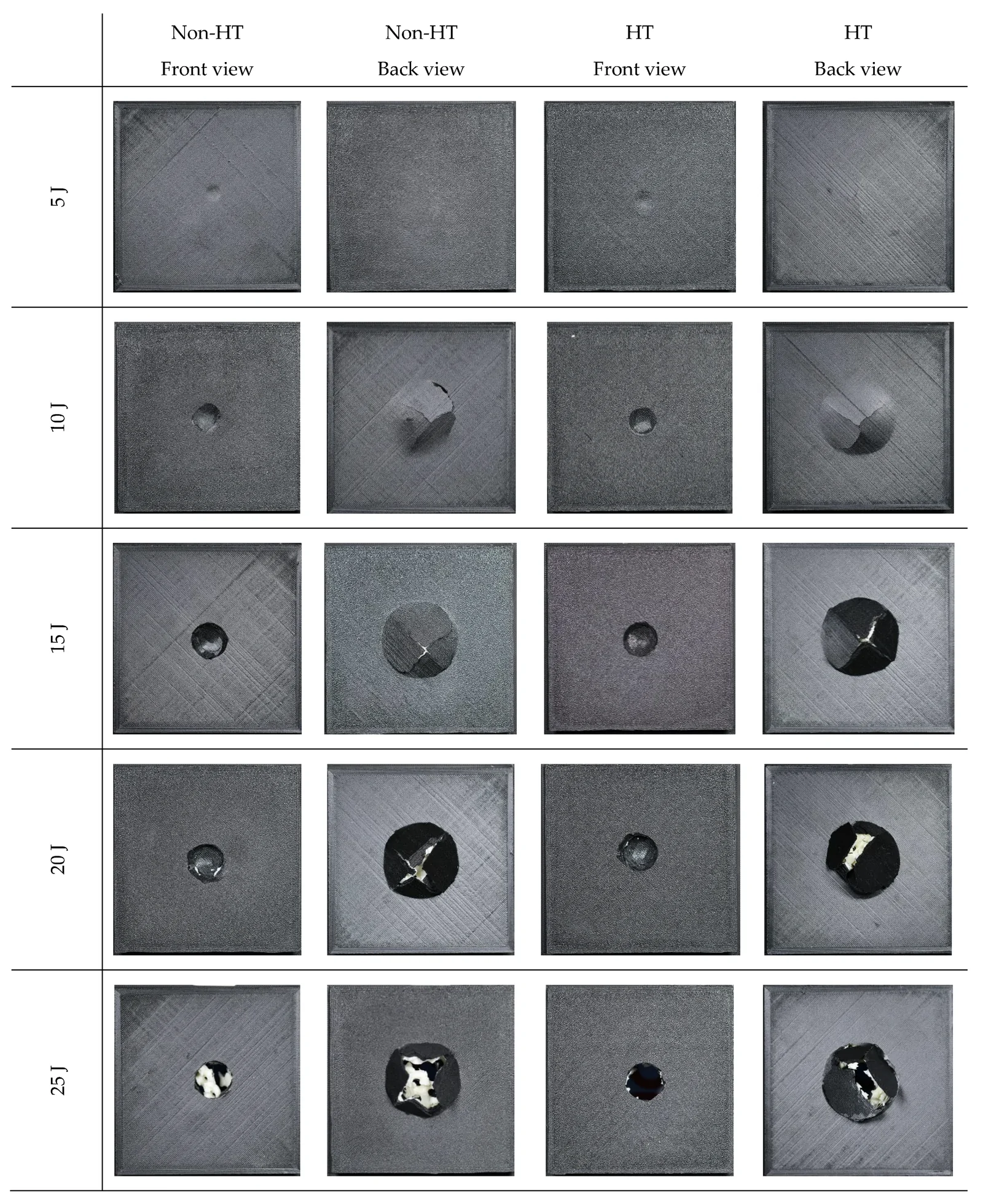
Impact damage morphology of the hybrid sandwich panels (PA6-CF face sheets with PA6 gyroid core) in the non-heat-treated (Non-HT) and heat-treated (HT) conditions; front and back views at impact energies of 5, 10, 15, 20, and 25 J.

Figure 13 shows the damage of the hybrid sandwich panels (PA6-CF face sheets, PA6 gyroid core) at 5–25 J in the Non-HT and HT conditions. At 5 J, only slight surface marks were observed, whereas from 10 J onward, local indentation formed on the front face and bending and opening occurred on the back face. At 15–20 J, front-face damage remained confined to the impact zone, while circular openings and radial cracks developed on the back face, indicating that damage progressed from the front face sheet through the core to the back face sheet. This progressive damage results from the combination of the stiff PA6-CF face sheets and the deformable PA6 core. Heat treatment did not fundamentally alter the general damage pattern, but it caused cracks to become sharper and more localized, particularly at energy levels of 15 J and above. At 25 J, both conditions showed severe front- and back-face damage at or near full penetration. At lower energies, the limited peak forces and long deformation times indicate controlled, progressive energy absorption rather than sudden brittle fracture.

**Figure 14:**
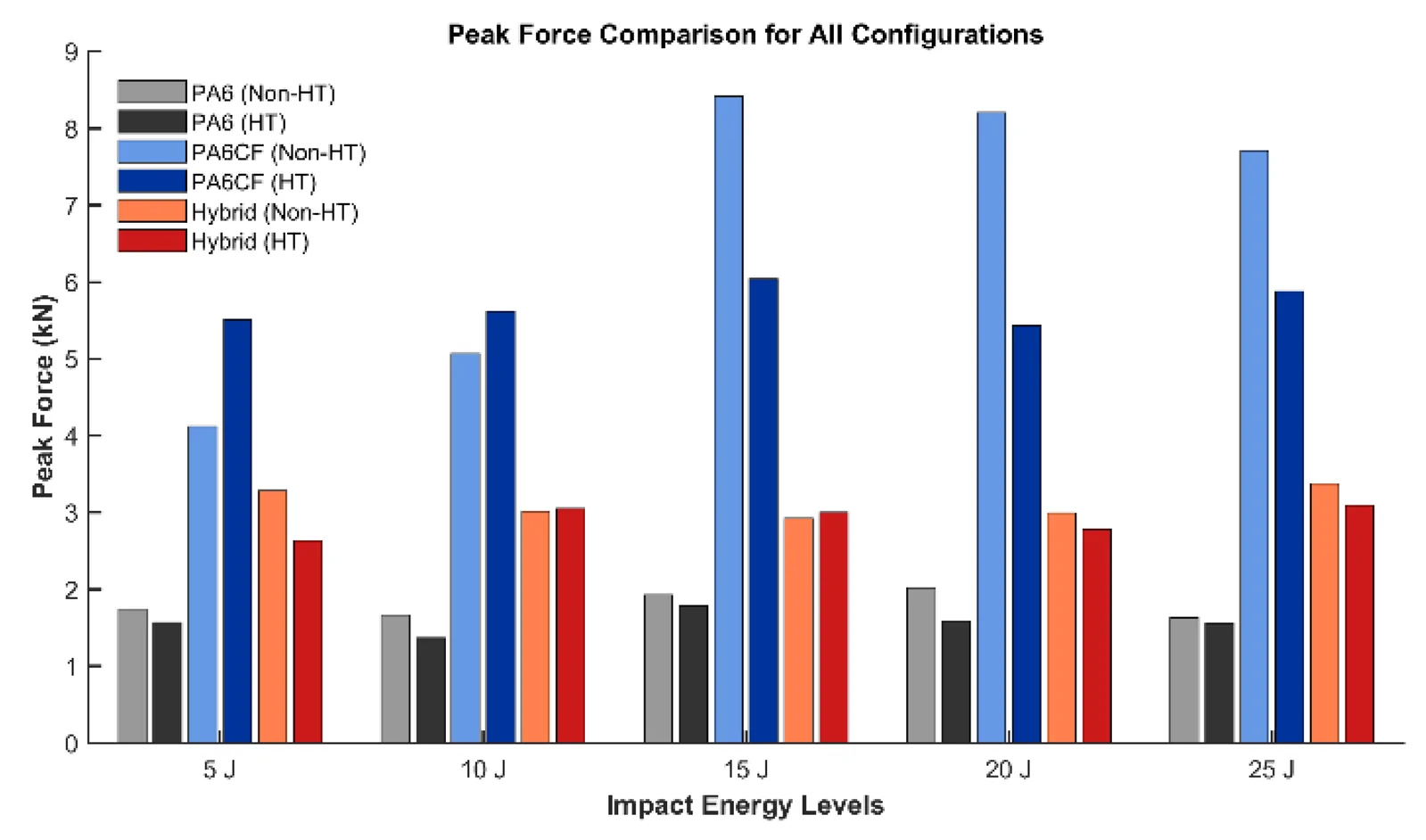
Comparison of peak contact forces across impact energy levels for all configurations.

Figure 14 compares the peak contact forces of all configurations as a function of impact energy. The PA6 specimens showed the lowest peak forces (approximately 1.4–2.0 kN) at all energy levels, and heat treatment caused only a slight decrease. The PA6-CF configuration showed markedly higher peak forces; in the Non-HT condition, the peak force increased from approximately 4.1 kN at 5 J to 8.4 kN at 15 J, and then decreased to approximately 8.2 and 7.7 kN at 20 and 25 J, respectively. The carbon fibers thus increase stiffness and load-carrying capacity, whereas at high energies, the peak force is limited by damage development. Heat treatment increased the peak force of PA6-CF at 5–10 J but reduced it at 15–25 J, by approximately 28% at 15 J and 34% at 20 J. The peak force of the hybrid panels remained within approximately 2.6–3.4 kN at all energy levels and was less sensitive to impact energy than that of PA6-CF.

**Figure 15:**
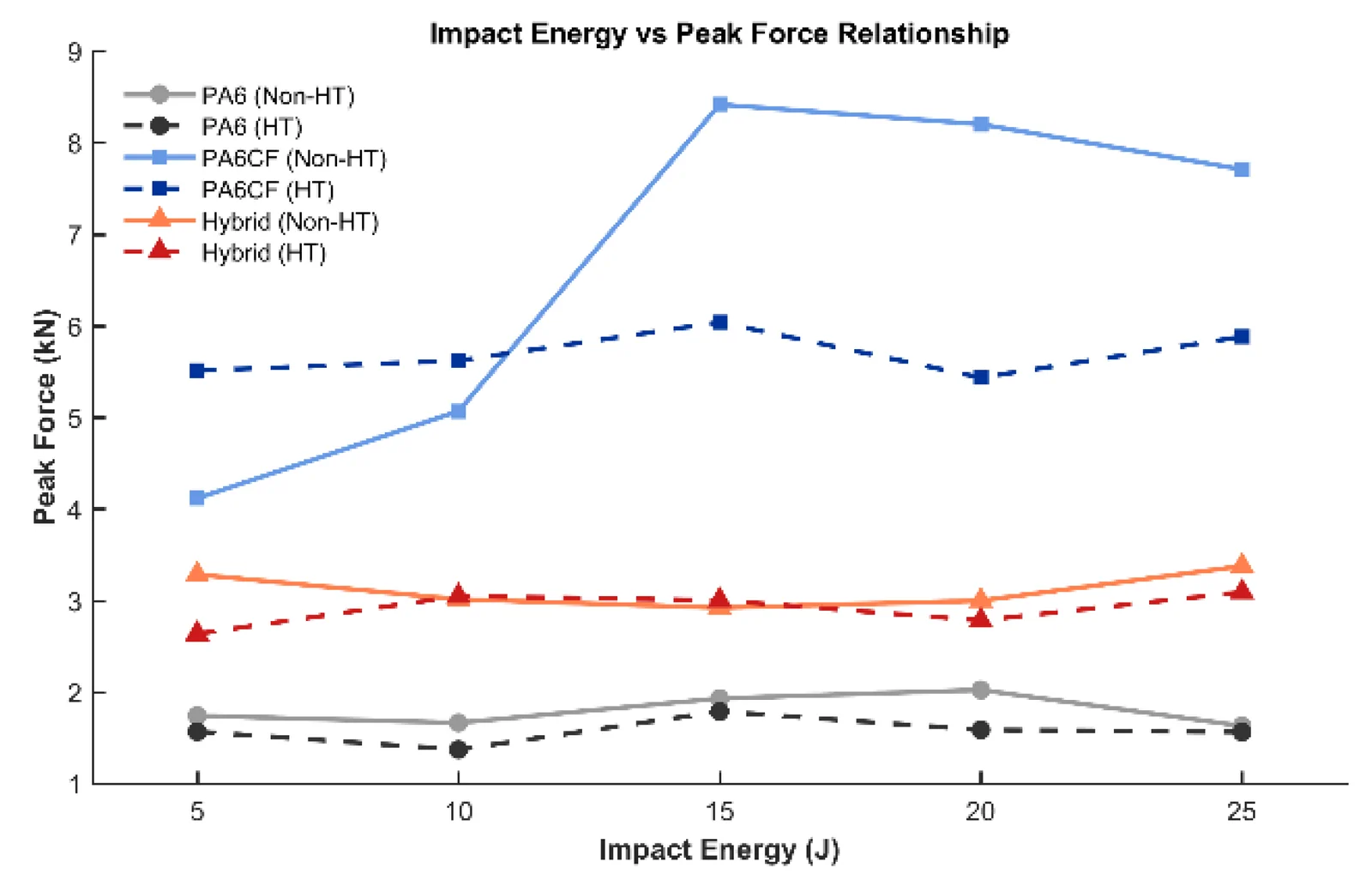
Impact energy versus peak force relationship as a function of configuration and heat-treatment condition.

Figure 15 shows the relationship between impact energy and peak contact force as a function of material configuration and heat treatment condition. The peak force of PA6 remained nearly constant over the entire energy range, indicating that the additional impact energy was accommodated by larger deformation rather than higher force. For Non-HT PA6-CF, the peak force increased sharply from 5 to 15 J and decreased above 15 J as damage progressed. Heat treatment markedly reduced the energy sensitivity of PA6-CF and stabilized the peak force within the range of approximately 5.5–6.0 kN. The hybrid panels varied only within approximately 2.6–3.4 kN in both conditions. PA6-CF therefore responds to increasing impact energy with high peak forces owing to its stiffness, whereas PA6 and the hybrid carry the load at lower forces through more controlled deformation.

**Figure 16:**
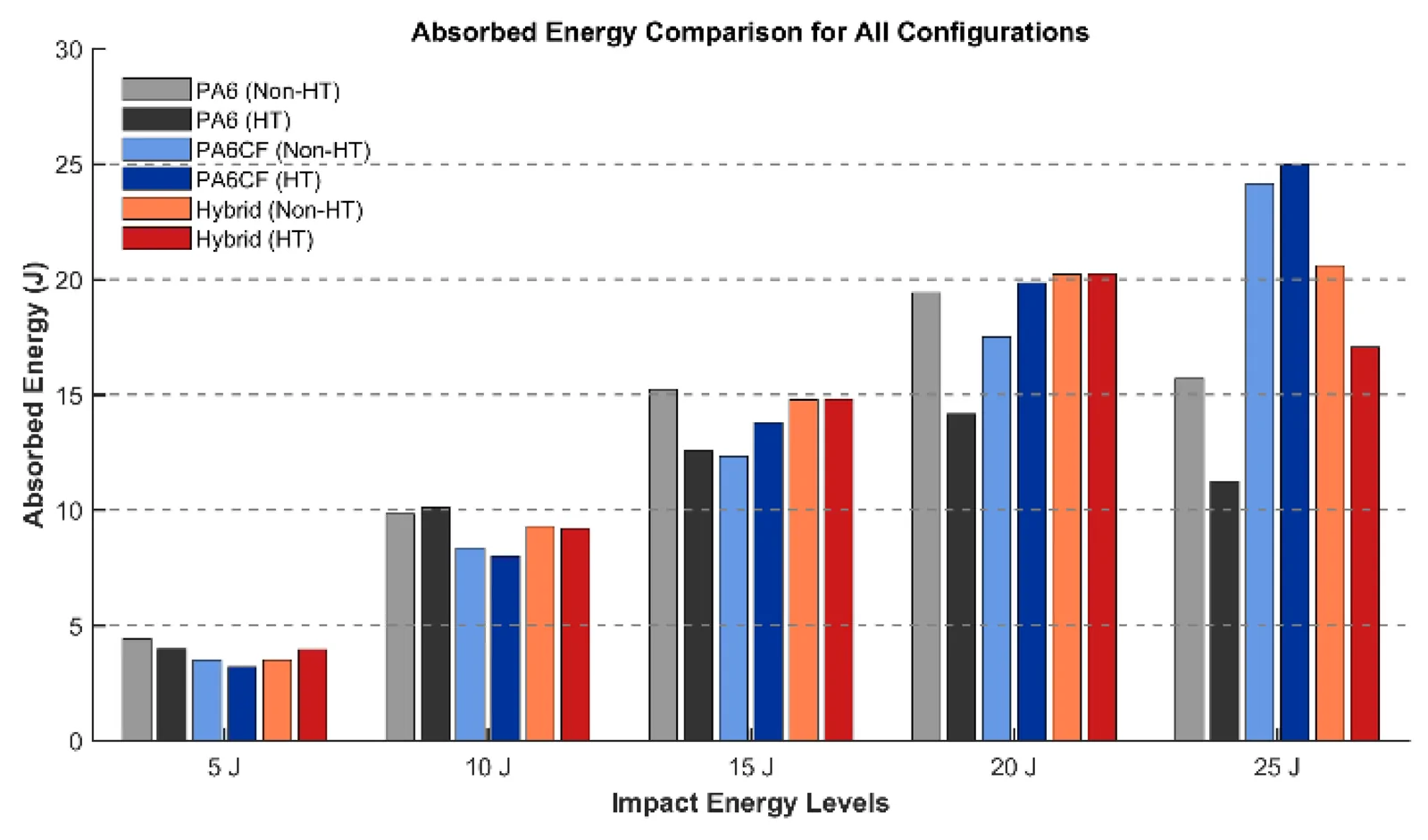
Comparison of absorbed energy across impact energy levels.

Figure 16 compares the absorbed energy of all configurations as a function of impact energy. The absorbed energy of Non-HT PA6 increased to 19.4 J at 20 J and dropped to 15.7 J at 25 J; after heat treatment, the values decreased to 14.2 and 11.3 J at 20 and 25 J, respectively. This decrease reflects the penetration and localized damage that limit the energy absorption of PA6 at high energies. The absorbed energy of PA6-CF increased steadily with impact energy and reached 24.2–25.0 J at 25 J. Heat treatment was beneficial for PA6-CF at high energies; the absorbed energy at 20 J increased from 17.5 J to 19.8 J, and at 25 J, it rose from 24.2 J to 25.0 J. The hybrid panels absorbed nearly all of the applied energy at 15 and 20 J (approximately 14.8 J and 20.2–20.3 J, respectively), with little difference between the HT and Non-HT conditions. At 25 J, the absorbed energy decreased to 20.6 J (Non-HT) and 17.1 J (HT). Up to 20 J, the damage of the hybrid panels remained contained without full penetration, and almost the entire impact energy was absorbed in both conditions; since the absorbed energy cannot exceed the impact energy, any difference between the HT and Non-HT panels was masked in this range. At 25 J, the damage approached full penetration, and once the front PA6-CF face sheet failed, the remaining energy had to be dissipated mainly by the PA6 core. As shown for the all-PA6 panels, heat treatment reduced the energy absorption capacity of PA6 at high energies (from 15.7 J to 11.3 J at 25 J), and the cracks in the HT hybrid panels became sharper and more localized (Figure 13). Therefore, the effect of heat treatment on the PA6 core became evident only when the damage reached the core and the back face sheet at 25 J, resulting in a lower absorbed energy in the HT condition. Heat treatment thus has opposite effects on PA6 and PA6-CF at high energies, reducing the absorbed energy of PA6 and increasing that of PA6-CF, while it has little effect on the hybrid up to 20 J.

**Figure 17:**
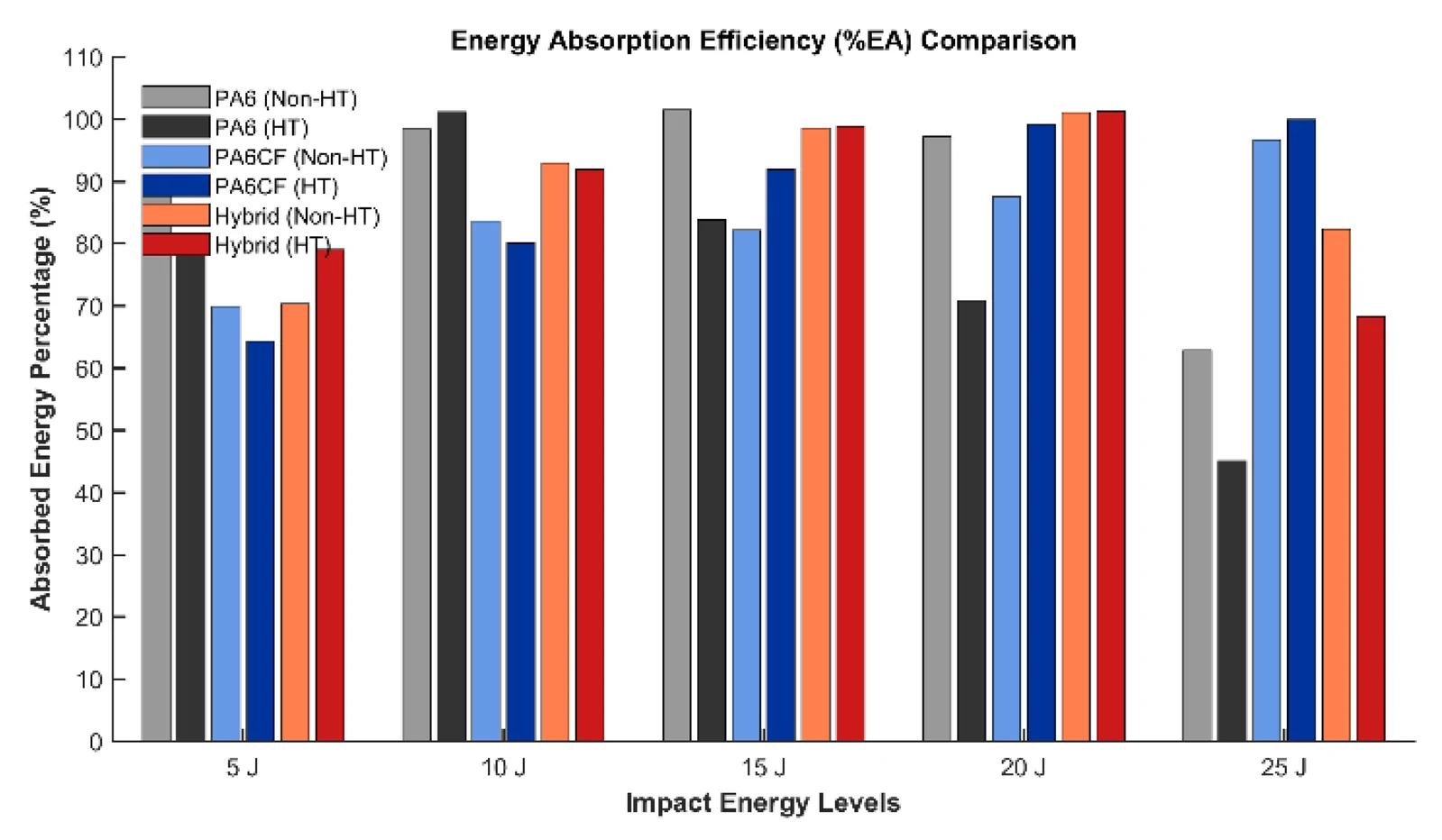
Comparison of energy absorption efficiency (%EA) across impact energy levels.

Figure 17 shows the energy absorption efficiencies (%EA) of PA6, PA6-CF, and hybrid sandwich structures at different impact energies. The efficiency of PA6 remained high at low and medium energies and decreased markedly at 20–25 J; at 25 J, the efficiency in Non-HT and HT specimens dropped to approximately 63% and 45%, respectively. This trend is consistent with the deformation capacity of PA6 being exceeded and penetration beginning at high energies. The efficiency of PA6-CF increased with impact energy, reaching approximately 97–100% at 25 J. Heat treatment increased the efficiency of PA6-CF at 15 J and above. The hybrid panels maintained a high efficiency of approximately 92–101% at 10–20 J, which decreased to approximately 82% (Non-HT) and 68% (HT) at 25 J as penetration progressed.

**Figure 18:**
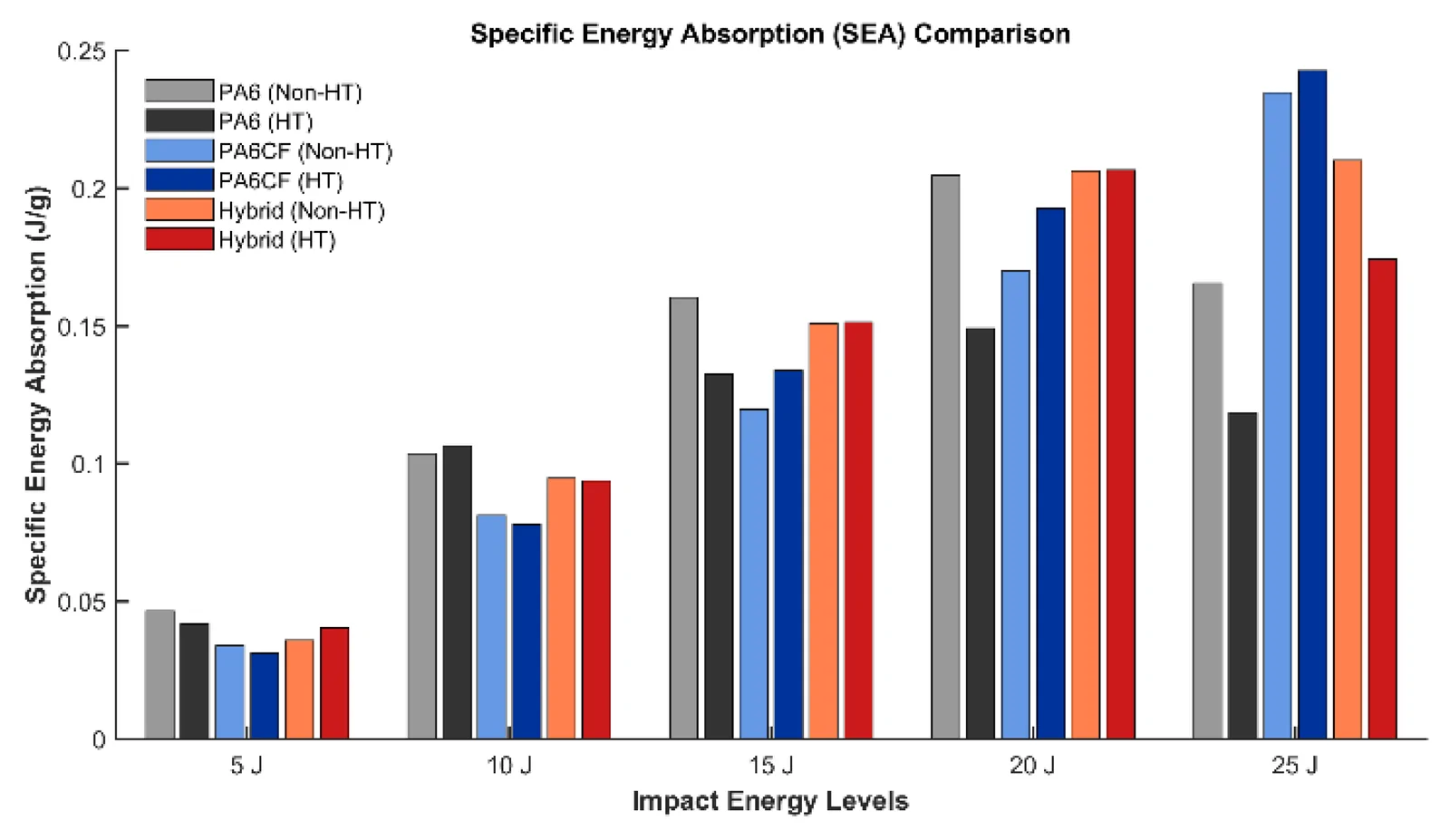
Comparison of specific energy absorption (SEA) across impact energy levels.

Figure 18 shows the specific energy absorption (SEA) values for all configurations at different impact energies. Differences between the configurations were small at low energies and increased with impact energy. The SEA of PA6 increased up to 20 J and decreased at 25 J, and heat treatment reduced the SEA of PA6 at 15–25 J. The SEA of PA6-CF increased continuously with impact energy, reaching a maximum of approximately 0.243 J/g at 25 J in the HT condition, despite the 8% higher mass of the PA6-CF panels. The hybrid panels reached approximately 0.15 and 0.21 J/g at 15 and 20 J, respectively, and decreased at 25 J, particularly in the HT condition. Because the panel masses differ by less than 8%, the SEA ranking follows the absorbed-energy ranking in Figure 16.

## 4. Conclusions

In this study, the effect of post-printing heat treatment (170 °C, 4 h) on the low-velocity impact behavior of PA6, PA6-CF, and hybrid (PA6-CF face sheets and PA6 gyroid core) sandwich structures produced by additive manufacturing was investigated in the energy range of 5–25 J. The main results are summarized below.

According to DSC analysis, heat treatment increased the degree of crystallinity of PA6-CF from 6.4% to 11.4%, while the increase in PA6 was limited (from 6.2% to 6.7%). This difference suggests that, although the carbon fibers retard crystallization during cooling from the melt, they leave a larger crystallizable fraction in the Non-HT PA6-CF matrix that develops during annealing; this difference in crystallinity may be one of the factors contributing to the contrasting impact responses of the two materials. However, on the basis of the present data, its contribution cannot be separated from those of residual-stress relief, drying, and changes in interlayer bonding.

The PA6-CF configuration exhibited the highest stiffness and peak forces; in the Non-HT condition, the peak force reached 8.41 kN at 15 J. Heat treatment reduced the peak force at 15 and 20 J by 28% and 34%, respectively, while increasing the absorbed energy at 20 J from 17.5 J to 19.8 J and yielding the highest SEA in this study (0.243 J/g at 25 J). Although the damage became more localized after heat treatment, the absorbed energy increased, which is attributed to energy dissipation during penetration, possibly through fiber–matrix interfacial damage and crack propagation.

In the unreinforced PA6 configuration, heat treatment had the opposite effect. The absorbed energy at 25 J decreased from 15.7 J to 11.3 J, the energy absorption efficiency dropped from 63% to 45%, and the damage was concentrated in a narrower area, indicating that heat treatment reduces the ductile energy absorption capacity of PA6.

The hybrid structure exhibited limited peak forces in the range of approximately 2.6–3.4 kN at all energy levels, an energy absorption efficiency of 92–101% in the 10–20 J range, and the lowest sensitivity to heat treatment. The combination of stiff PA6-CF face sheets with a deformable PA6 core resulted in controlled, progressive damage rather than sudden brittle fracture. As penetration progressed at 25 J, the efficiency decreased to 82% in the Non-HT condition and to 68% in the HT condition.

The findings indicate that post-printing heat treatment is an effective parameter for adjusting the impact response without changing the geometry or material; however, the direction of its effect depends on the presence of fiber reinforcement and the impact energy. Therefore, heat treatment conditions must be selected specifically for the material configuration. Since the HT route included a vacuum-drying step before annealing, the observed differences reflect the combined effect of drying and annealing; the separate contribution of moisture content could not be quantified in the present study. Investigating different annealing temperatures and durations, core relative densities, and repeated impact scenarios, moisture-matched Non-HT controls with moisture-content measurements before testing, as well as SEM fractographic analysis to confirm the proposed micro-scale damage mechanisms, is recommended for future studies.

## Figures and Tables

**Table 1:** Technical properties of the PA6 and PA6-CF filaments [68].

Technical Property	PA6	PA6-CF
Density	1.13 g/cm^3^	1.24 g/cm^3^
Build Plate Temperature	80–100 °C	80–100 °C
Nozzle Temperature	240–260 °C	265–290 °C
Tensile Strength	80 MPa	120 MPa
Elongation at Break	4%	2%

**Table 2:** Single-screw filament extrusion parameters.

Parameter	PA6	PA6-CF
Feed Zone	215 °C	215 °C
Melting Zone	225 °C	225 °C
Transfer Zone	225 °C	225 °C
Die Zone	222 °C	222 °C
Screw Speed	25 rpm	32 rpm
Haul-off Speed	33 m/min	43 m/min

**Table 3:** Material configuration of the test specimens.

Layer	Hybrid	PA6-CF	PA6
Top Plate	PA6-CF	PA6-CF	PA6
Gyroid Core	PA6	PA6-CF	PA6
Bottom Plate	PA6-CF	PA6-CF	PA6

**Table 4:** Printing parameters.

Parameters	PA6-CF	PA6
Build Plate Temperature	100 °C	100 °C
Nozzle Temperature	290 °C	260 °C
Part Cooling Fan	Closed	Automatic
Chamber Cooling Fan	0%	60%
Printing Direction	Rectilinear	Rectilinear
Printing Speed	80 mm s^−1^	100 mm s^−1^
Layer Height	0.2 mm	0.2 mm
Nozzle Diameter	0.4 mm	0.4 mm
Chamber Temperature	60 °C	60 °C

**Table 5:** DSC analysis results of PA6-Non-HT, PA6-HT, PA6-CF-Non-HT and PA6-CF-HT specimens.

	Tc (°C)	Tm (°C)	∆*H_m_* (J g^−1^)	Xc (%)
PA6-Non-HT	198	234	14.3	6.2
PA6-HT	198	237	15.5	6.7
PA6-CF-Non-HT	184	233	12.5	6.4
PA6-CF-HT	184	231	22.3	11.4

**Table 6:** Impactor mass and impact velocity corresponding to each impact energy level.

Impactor Mass (kg)	Impact Energy (J)	Impact Velocity (m s^−1^)
5.295	5	1.374
	10	1.943
	15	2.380
	20	2.749
	25	3.073

## Data Availability

The raw data supporting the conclusions of this article will be made available by the authors on request.
